# Small-molecule inhibitors of 6-phosphofructo-1-kinase simultaneously suppress lactate and superoxide generation in cancer cells

**DOI:** 10.1371/journal.pone.0321998

**Published:** 2025-05-21

**Authors:** Samo Lešnik, Janez Konc, Tina Vodopivec, Katja Čamernik, Urška Karolina Potokar, Matic Legiša

**Affiliations:** 1 Faculty of Chemistry and Chemical Engineering, University of Maribor, Maribor, Slovenija; 2 Department of Molecular Modeling, National Institute of Chemistry, Ljubljana, Slovenia; 3 Biotechnical Faculty, University of Ljubljana, Ljubljana, Slovenia; 4 Department of Synthetic Biology and Immunology, National Institute of Chemistry, Ljubljana, Slovenia; University of Nairobi, KENYA

## Abstract

Deregulated energy metabolism is a hallmark of cancer, characterized by increased glycolytic flux. Cancer-specific modification of 6-phosphofructo-1-kinase (PFK) impairs its ability to regulate the enzyme’s activity which increases glycolytic flux. Consequently, excessive cytosolic NADH formation triggers a harmful redox imbalance in cancer cells, which is rapidly neutralized by the formation of lactic acid and superoxide (SOX). To learn more about deregulated glycolysis in cancer cells, a supercomputer used the atomic model of the crystal structure of human PFK1 for virtual screening a database of 4.5 million compounds by docking with the catalytic binding sites of the enzyme. The screening revealed two compounds capable of reducing modified, cancer-specific PFK1 activity and simultaneously suppressing lactate and SOX formation. A dose-dependent inhibition was observed in the cells treated by compounds in the following tumorigenic cells: Jurkat (Acute T cells leukemia); Caco-2 (colorectal adenocarcinoma); COLO 829 (melanoma); and MDA-MB-231 (breast gland adenocarcinoma). In addition, two selected compounds assessed for cytostatic and cytotoxic activity showed no negative effects on tumorigenic cells. However, during incubation, the strengths of inhibitions continuously decreased, both during lactate and SOX formation. No such effects were observed if compounds were sequentially submitted to the cells at low concentrations every 24 hours. Additional experiments performed by Jurkat cells revealed reduced respiration and glycolysis rates in the cells treated with compounds concerning the untreated cells. Inhibition of modified cancer-specific PFK1 activity reduces deregulated glycolytic flux, prevents abundant cytosolic NADH formation, and restores redox balance thus simultaneously preventing the formation of deleterious effects of lactate and SOX, two crucial players in cancer initiation and development.

## Introduction

Cancer is a disease characterized by uncontrolled cell proliferation, whereas in normal, differentiated cells, growth and replication are tightly controlled by growth factors and signal transduction. To maintain their growth, cancer cells undergo complex restructuring characterized by changes in metabolic pathways involved in energy production and biosynthetic processes. One of these changes, designated a “hallmark of cancer” [1], is the deregulation of cellular energetics.

There are reasons to believe that the post-translational modification of 6-phosphofructo-1-kinase may cause aberrant energy metabolism in cancer cells [2]. The native human 6-phosphofructo-1-kinase (PFK1) (EC 2.7.1.11) exhibits the most complex control of the glycolytic pathway in normal cells, surpassing the regulatory functions of other allosteric enzymes. PFK1 catalyzes the phosphorylation of fructose-6-phosphate to fructose-1,6-bisphosphate using Mg-ATP as a phosphoryl donor [3]. PFK1 is stimulated by fructose-2,6-bisphosphate (F-2,6-BP), ADP/AMP, and ammonium ions, whereas citrate and ATP act as potent inhibitors [4].

Three different PFK1 genes are present in the human genome: muscle type (PFK-M), 85.183 Da [5]; liver type (PFK-L), 85.018 Da [6]; and platelet type (PFK-P), 85.596 Da [7], which are expressed differently in specific tissues. Different levels of isoenzymes have also been found in tumor-forming cells [8] and may differ in certain stages of growth in individual tumors [9].

During evolution, eukaryotic PFK1 enzymes developed by duplication, tandem fusion, and divergence of a prokaryotic ancestor’s catalytic and effector binding sites [10]. However, the strict conservation between the active site residues in the N-terminal segment of the eukaryotic enzyme and those of bacterial PFKs suggests that the active site of eukaryotic PFK1 is located in the N-terminal part [10]. In contrast, the allosteric ligand binding sites, which have arisen during evolution through mutations in the C-terminus, allow fine-tuning of the regulatory enzyme through the increased levels of specific downstream metabolites. One of the allosteric ligands is citrate, which acts as a potent inhibitor of all mammalian PFK1 isoforms [11], while some effectors, such as fructose 2,6-bisphosphate, increase the activity of the enzyme to a higher level than that of the native enzyme. The native 87-kDa PFK1s is, therefore, a key regulatory enzyme of glycolysis.

We were the first to show that in cancer cells, the human native PFK1 enzymes, which are generally under the control of feedback inhibition, undergo post-translational modifications. After proteolytic cleavage of the C-terminal part of the enzyme, highly active, shorter 45-47 kDa in PFK-M isoenzyme [2], and 70 kDa in PFK-L isoenzymes fragments [12] are formed that are insensitive to citrate and ATP inhibition. However, no post-translational modification of PFK-P isoform has been observed in MDA-MB-231 cells [13] or other tumorigenic cells.

Deregulated cancer-specific, highly active PFK-M, and PFK-K fragments lacking allosteric binding sites trigger uncontrolled glycolytic flux and abundant cytosolic nicotinamide adenine dinucleotide (NADH) generation as a by-product of glycolysis. This occurs at the sixth step of glycolysis acolytes where two molecules of glycerol-3-phosphate are oxidized to two molecules of 1,2-bisphosphoglycerate coupled with the reduction of NAD^+^ to NADH by glyceraldehyde-3-phosphate dehydrogenase (GAPDH) [14].

The balance between NADH/NAD^+^ and NADPH/NADP^+^ (nicotinamide adenine dinucleotide phosphate) is vital in all organisms. Both pyridine nucleotide molecules act as freely diffusible electron carriers; however, they are engaged in distinct metabolic pathways. NADH drives energy production in the cytosol by glycolysis and in the mitochondria by oxidative phosphorylation, while NADPH governs reductive biosynthesis, antioxidation, and oxidative stress [15]. To maintain redox homeostasis in proliferating cells, redundant cytosolic NADH must be re-oxidized by two mechanisms; in the cytosol, NADH is re-oxidized by reducing pyruvate as the last step of glycolysis, into lactic acid, which is finally transported out of the cells [16]. The effect of deregulated metabolic flux, redox imbalance, and abundant NADH formation was observed in *pfk*-null *S. cerevisiae* hosting recombinant highly active cancer-specific PFK-M enzyme [17].

The extracellular accumulation of lactic acid leads to acidification of the tumor microenvironment, creating a pH gradient between the extracellular pHe (6.6-6.9) and the intracellular pHi (7.2-7.5), which supports the malignant phenotype [18] characterized by immune evade [19], metastases [20], triggering angiogenesis [21], and inflammatory responses [22].

Simultaneously the malate-aspartate shuttle translates some redundant cytosolic NADH into the mitochondrial compartment [23]. When NADH molecules need to be re-oxidized (each NADH molecule donates one pair of electrons), the electrons are passed to the mitochondrial electron transport chain (ETC). Still, some escape permanently from the ETC and react directly with oxygen to generate Superoxide ions (O_2_^-^.) Most commonly, superoxide (SOX) is formed at cytochrome III level by incomplete one-electron reduction of oxygen [24]. SOX is short-lived but can be rapidly converted into other Reactive oxygen species (ROS). By superoxide dismutase hydrogen, peroxide (H_2_O_2_) is formed, which is more stable and can diffuse throughout the cell. Increased steady-state cytosolic concentrations of SOX may also reduce transition metals, which in turn react with H_2_O_2,_ producing hydroxyl radicals (OH•) [25]. Hydroxyl radicals are strong oxidants capable of irreversible nitration of proteins, inactivating enzymes, and causing DNA lesions [26].

In the past, numerous attempts were made to suppress the individual synthesis of lactate or SOX by inhibiting various metabolic targets. However, suppressing one method of NADH re-oxidizing must inevitably increase another system of re-oxidation to maintain appropriate redox balance. Using small-molecule inhibitors to suppress cancer-specific PFK1 activities to the level of normal PFK1 cells, seems to be the more effective method for reducing deregulated glycolysis flux.

**Fig 1:**
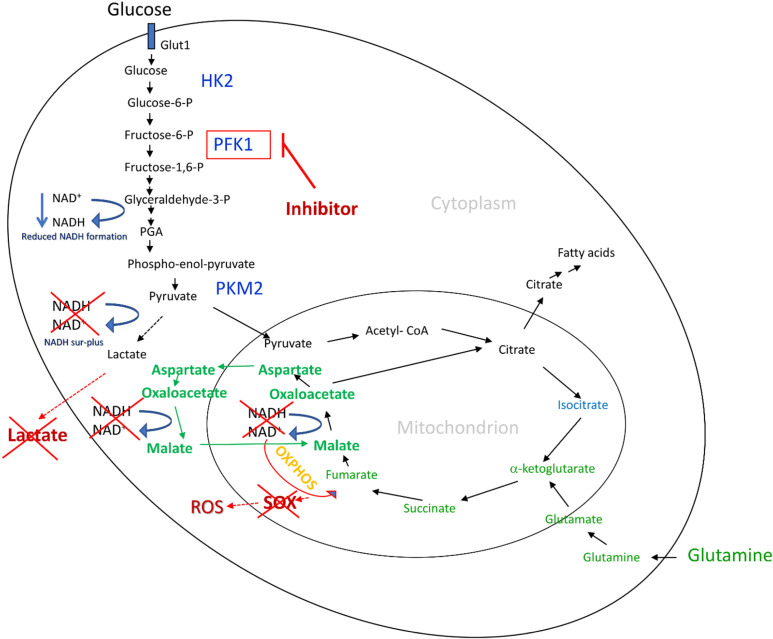
A schematic presentation of simultaneous prevention of lactate and ROS/SOX generations by using small-molecule compounds to reduce the activity of cancer-specific 6-phosphofructo-1-kinase (PFK1). Dysregulated metabolic processes in cancer lead to the formation of numerous glycolytic intermediates, including cytosolic NADH. As abundant NADH is formed at the level of glyceraldehyde-3-phosphate, the cellular redox system becomes unbalanced. Excess NADH must be reoxidized immediately to avoid damaging disruption through lactate and superoxide formation. While NADH is mainly reoxidized in the cytosol by reducing pyruvate to lactate, part of the NADH enters the mitochondrial compartment through the aspartate-malate shuttle. Mitochondrial NADH is oxidized by donating electrons to the electron transport chain (ETC) in oxidative phosphorylation (OXPHOS). If there is an excess of electrons, some can incompletely reduce the oxygen with a single electron and form superoxide. Small molecule cmpds of 6-phosphofructo-1-kinase (PFK1) reduce the activity of the enzyme’s highly active, cancer-specific form to the level of normal PFK1 enzymes. At the same time, the metabolism in the cancer cells is reduced, and the formation of abundant NADH is suppressed. Restoring the redox balance eliminates the need for NADH reoxidation, and prevents lactate and superoxide formation.

This study shows that selected small-molecule inhibitors discovered to reduce modified cancer-specific PFK1 activities are suitable means for inhibiting dysregulated glycolytic flux in cancer cells simultaneously preventing lactate and SOX formation. The reduced glycolytic flux avoids excess cytosolic NADH formation and shows no cytostatic and cytotoxic effects. Besides, by suppressing lactate and SOX formation detrimental developmental effects such as angiogenesis, immune evade, metastases, and mutations are prevented (Fig 1).

## Materials and methods

### PFK1 molecular modeling and small-molecule inhibitors screening

To find the drugs inhibiting highly active, cancer-specific PFK1s, the atomic model of the human PFK-P iso-enzyme was designed based on the crystal structure of the human PFK-P tetramer (UniProt Q01813) in combination with ATP–Mg^2+^ at a resolution of 3.1 Å (Protein Data Bank accession number 4XYJ) [27]. In collaboration with the Laboratory for Molecular Modelling at the National Institute of Chemistry, Ljubljana, the ZINC Drug NOW database was first filtered to exclude expected aggregators and poorly soluble compounds. By using a supercomputer (CROW 16, National Institute of Chemistry, Ljubljana, Slovenia) with approximately 3,000 processor cores, large-scale virtual screening was performed by docking with ProBiS-Dock algorithm [28,29] to the catalytic ATP binding site of PFK-P/PFK-M and PFK-L isoenzymes.

### Tumorigenic cell lines

The tumorigenic cell lines Jurkat (acute T cell leukemia; TIB-152), Caco-2 (colorectal adenocarcinoma; HTB-37), COLO 829 (melanoma; CRL-1974), were purchased from American Type Culture Collection (Manassas, Virginia, USA) while MDA-MB-231 cells (breast gland adenocarcinoma; CRM-HTB-26) were obtained through the courtesy of investigators at Josef Stefan Institute (Ljubljana, Slovenia). Mycoplasma testing was performed on all cell lines before being used in the experiments.

### Cellular and metabolic analyses

If not specified differently, the tumorigenic cells were plated in the RPMI 1640 GlutaMax medium (Thermo Fisher Scientific, Waltham, MA, USA) supplemented with 10% FBS at the concentration of 5 × 10^5^ cells per mL. The cells were incubated at 37^o^C and 5% CO_2._ Different concentrations of specific inhibitors were added to the medium at the beginning of inoculation. An adequate amount of DMSO used in the solution with the specified concentrations of compounds was added to the vehicle.

#### Lactic acid measurements.

For measuring lactate concentrations in the medium, 20 µL samples were taken at specified time intervals, centrifuged at 1200 rpm for 5 minutes at 4^o^C and 15 µL supernatants were preserved at 4^o^C until analyzed.

Lactate levels were assessed enzymatically using an L-Lactic acid assay kit (K-LATE, Megazyme, Bray, Ireland) and measured according to the manufacturer’s instructions.

#### SOX/ROS measurements.

For measuring intracellular levels of reactive oxygen species tumorigenic cell lines were initially grown in the RPMI 1640 GlutaMax medium, as described above. After 78 hours cells were washed by centrifugation, and an aliquot of 1 × 10^5^ cells was placed into 1 mL of phenol red-free IMDM medium (Thermo Fisher Scientific, Waltham, MA, USA) supplemented with 10% of FBS. The cells were incubated for an additional 3 hours before measurement.

For detecting ROS and SOX levels in the cells, 100 µL of medium was washed with PBS by standard centrifugation protocol and then placed into a well on the 96-well black wall/clear bottom plate. To each well 100 µL ROS detection reagent (Green) and SOX detection reagent (Orange) were added as specified in the Cellular ROS/SOX Detection Assay Kit (ab139476 Abcam, Cambridge, UK). For measuring superoxide Cellular Superoxide Detection Assay Kit (ab139477 Abcam, Cambridge, UK) was used that contains “Orange” but not the “Green” reagent. Relative fluorescence was measured by a microplate reader (Biotek, Vermont, USA), using Ex/Em 525 nm for the “Green” and Ex/Em 550/620 for the “Orange” reagent. For the positive control ROS inducer Pyocyanin was used and for the negative control ROS inhibitor N-acetyl-L-cysteine was used according to the manufacturer’s instructions.

### Cytostatic and cytotoxic assays

#### Cytostatic assay.

Total Cell numbers at the end of incubation (72 hours) were determined by the XTT cell proliferation assay Kit (Cat. No. 30-1011K, ATCC, Manassas, Virginia) according to the manufacturer’s instructions. Untreated cells and cells treated with different concentrations of inhibitors were incubated for 72 hours before alive cellular biomass was determined according to the manufacturer’s instructions.

#### Cytotoxicity.

Cell viability was determined by the Cytotoxicity Detection Kit (LDH) (Cat. No. 11 644 793 001, Sigma-Aldrich, Steinheim, Germany) according to the manufacturer’s instructions. As a negative control, 100 µL Triton X-100 was added to wells containing 1mL medium with cells. For the survival evaluation in the dose-dependent tests, the measurements were conducted at the end of incubation at 72 hours of growth in the medium.

### Measurements of respiratory and glycolytic rates and capacities

The efficiency of PFK1 inhibitors was evaluated by measuring respiratory and glycolytic rates and capacities in treated and untreated Jurkat cells. For this experiment, the Seahorse XFp instrument (Agilent Technology, Santa Clara, CA, USA) was used under standard conditions before and after the addition of 1 µM of oligomycin A and 0.25 µM of Carbonyl cyanide-4**-(**trifluoromethoxy)phenylhydrazone (FCCP). The cells were incubated in RPMI 1650 GlutaMAX medium and inoculated with inhibitors No. 9 and No. 30 at the final concentration of 10 µM. An adequate amount of DMSO was added to the vehicle. After 24 hours of growth, the cell numbers were normalized to (3.33x10^6^ per mL). For the experiment 400 µl culture was washed by centrifugation and placed into XF Base medium with added glucose (10 mM), glutamine (2 mM), and Na-pyruvate (1 mM). A day before the experiment the Kit boxes were poured with poly-l-lysine (50 µL) (cell sticker) which was removed before the medium with Jurkat cells was added. The measurements were conducted essentially as described in the Seahorse XFp protocol. After 20 minutes of incubation respiration and glycolytic basal rates were measured, followed by adding 1 µM of oligomycin A (ATP synthase inhibitor) and 0.25 µM of FCCP (mitochondrial oxidative phosphorylation uncoupler). After 6.5 minutes OCR and ECAR values were determined again to compute maximal respiration and glycolytic capacity.

### Co-culture of tumorigenic Jurkat cells and activated T-cells

#### Isolating immune cells.

Peripheral Blood Monocyte Cells (PBMCs) were donated by Dr. V. Forstnerič, a colleague in the lab. Samples were obtained with informed consent and according to the study protocol approved by the National Medical Ethics Committee (0120-21/2020/4). Depletion of non-T cells from the PBMCs was conducted by Ficoll® Paque gradient centrifugation followed by Pan T Cell Isolation kit according to the manufacturer’s instructions (Miltenyi Biotec GmbH, Auburn, CA). Consequently, unharmed T-cells were isolated containing levels of enriched CD4 helper T-cells and CD8 cytotoxic T-cells. Isolated T-cells were grown in 12 well plates containing 1 mL medium. Growth stimulating medium contained in a total volume of 50 mL, X-Vivo 15 medium (Lonza, Basel, Switzerland) plus 5% FBS; 6.25 µL of 80 IU/mL IL-2; 70 µL/mL ImmunoCult^TM^ human CD3/CD28 T-cell activator (Stemcell Technologies, Vancouver, Canada); and 0.17 µL 5 µM 2-mercaptoethanol. The T-cells were seeded in the medium at a concentration of 5.10^5^ cells per mL. After 3 days of incubation when the number of stimulated cells reached 2–3.10^6^ cells per mL, DMSO was added to the final concentration of 10%, and the activated T-cells were stored at -80^o^C until use.

#### The combined growth of Jurkat cells and activated T-cells.

Before the start of the co-culture, the Jurkar cells were pre-grown in RPMI 1640 GlutaMax medium without sodium bicarbonate to enable pH alternation (Product number R6504, Sigma-Aldrich, Steinheim, Germany) with added FBS to the final concentration of 10% and incubated as specified above. The specific inhibitors were added to 10 µM concentration and sequentially reintroduced at 24-hour intervals. The tests started with 1 × 10^5^ cells per mL. After 72 hours of Jurkat cell growth, immediately after the sequential reintroduction of inhibitors, the cells were collected with centrifugation, and the supernatant was saved for further experiments. An aliquot of precipitated Jurkat cells was added to 1mL of pre-used supernatant to reach a final cell number of 1.10^4^ cells per mL. The pre-used medium has been taken to maintain the pH value of the medium during the co-culture experiment. Finally, to each well with Jurkat cells an aliquot of activated T-cells was added (5.10^4^ per mL). Before measuring the co-culture was incubated under standard conditions for 18 hours.

#### Measuring apoptosis in a co-culture of Jurkat cells and activated T-cells.

Early and late-stage apoptosis of Jurkat cells in a co-culture were detected after washing cells with PBS and adding fluorescently labeled apoptotic dye Annexin V and viability dye 7-AAD. eBioscience Annexin V Apoptosis Detection Kit eFluor 450 (Cat. No 88-8006) (Thermo Fisher Scientific, Waltham, MA, USA) was used for all experiments according to the manufacturer’s instructions.

Fluorescence was measured using a Spectral Flow Cytometer (Aurora Cytometer, Cytek Biosciences, Amsterdam, The Netherlands) and analyzed using FloJo software (Tree Star Inc., San Carlos, CA, USA). Annexin V+/7-AAD+ (late apoptotic) and annexin V+/7-AAD- (early apoptotic) cells were quantified by the frequency of fluorescently labeled cells and statistical significance was assessed by the two sample T-test (independent variable).

### Statistics

Data are representative of three independent measurements and are presented as means ±(SD) (n = 3). Calculations from three independent experiments were performed with evaluation of statistical significance using two-tail Student’s T-tests (Graph Pad Prism version 3.0 (Graph Pad Software).

## Results

### Selected compounds

In the human PFK1 isozymes, two types of binding sites characteristic of a) the PFK-P/PFK-M isoform and b) the PFK-L isoform were used for virtual screening of the ZINC Drug NOW database on a supercomputer. Of the 38 compounds chosen by the screening, 33 were commercially available, 18 were selected by docking to the PFK-P/PFK-M type of ATP catalytic binding site, and 15 were more likely to bind to the PFK-L type of ATP binding site.

### Preliminary screening of selected compounds for suppressing lactate and superoxide (SOX) formation in four tumorigenic cell lines

First, all selected compounds (33) were tested for suppressing lactate and SOX by 4 tumorigenic cell lines. In the experiment, tumorigenic cell lines were inoculated with 50 µM of the respective potential inhibitors at an initial concentration of 1 × 10^6^ cells and incubated for 36 hours. The concentrations of extracellular lactate and mitochondrial SOX were determined as described in the Materials and Methods. The statistically significant difference between untreated cells (DMSO control vehicle) and cells treated with each inhibitor was determined.

**Fig 2:**
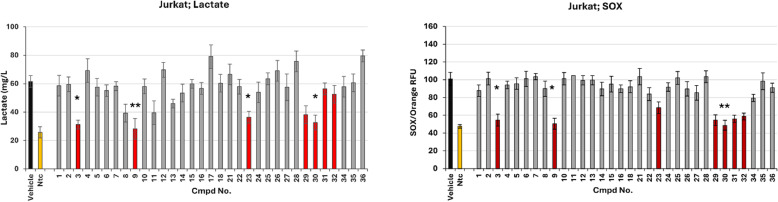
Preliminary screening of selected compounds for suppressing lactate and superoxide formation in Jurkat cells. First, all 33 commercially available compounds selected by screening were examined to determine whether they suppress the formation of lactate and mitochondrial superoxide (SOX) by Jurkat cells. After 36 hours of incubation, statistically significant differences were observed between untreated (vehicle) and treated cells exposed to 50 µM of each inhibitor. In the Jurkat cells, four cmpds were found to suppress lactate (P* < 0.005), compared to the vehicle. However, the lowest level of significant difference in lactate suppression between treated and untreated cells was observed by cmpd No. 9, (P** < 0.001). By measuring SOX suppression, cmpd No. 30 proved to be the most successful (P** < 0.001), while cmpds No. 3 and 9 showed slightly higher values (P* < 0.005), compared to the vehicle. All compounds that showed a more potent inhibitory effect on a tumorigenic cell are marked in red.

**Jurkat cells** showed the lowest value of significant difference in lactate suppression compared to untreated cells with cmpd No. 9, while cpmds No. 3, 23, and 30 showed higher values. Similar results were obtained for the suppression of SOX formation, with cmpds No. 30 showing the most significant difference, while cmpds No. 3 and 9 were less effective. No significant differences were observed between vehicles and cells treated with other compounds for the lactate or SOX generations (Fig 2).

In **Caco-2 cells**, the most striking statistical differences were found in suppressing lactate by cmpds No. 3, 9, 23, and 30, while the most potent suppression of SOX formation was by cmpds No. 3, 9, 30, and 32. No significant differences were observed in either lactate or SOX formation between the vehicle and cells treated with other compounds (S1 Fig).

Lactate formation was also significantly suppressed in **COLO 829 cells** when treated with cmpd No. 3, 9, 29, and 30. However, cmpd No. 9 showed the most significant differences. The cmpds No. 3, 9, 23, and 30 showed significant differences in SOX suppression between vehicle and treated cells. No significant differences were observed between vehicles and cells treated with other compounds for either lactate or SOX generations (S2 Fig).

Significant differences in lactate suppression in **MDA-MB-231 cells** between vehicle and cells treated with cmpds No. 3, 9, or 30 were observed, with cmpds No. 9 and 30 showing more decisive differences. Cells treated with cmpd No. 30 also showed the lowest P values for suppression of SOX formation; however, they were slightly reduced, but significant abilities to suppress SOX formation were observed in the presence of cmpds No. 3 or 9. No significant differences were observed in either lactate or SOX formation between the vehicle and cells treated with other compounds (S3 Fig).

**Fig 3:**
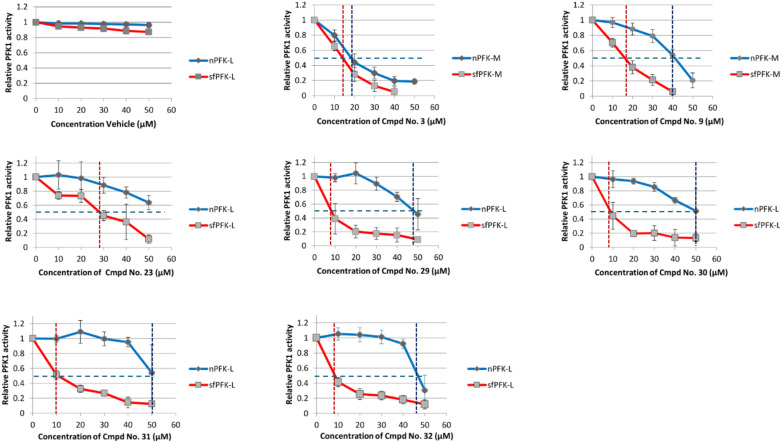
Half maximal inhibitory concentrations (IC_50_) of selected compounds. In the graphs, the red horizontal lines present the intersections among the concentrations of modified sfPFK-M enzymes, while horizontal blue lines are from native nPFK-M enzymes. The vertically dashed line shows the half-maximal PFK-M activities of enzymes treated by different concentrations of selected compounds. Selected compounds were evaluated for their ability to reduce purified human PFK1 enzyme activities in vitro. Cmpds No. 3 and 9, which preferentially bind to the ATP-binding site of PFK-M, inhibited both the native (85 kDa) enzyme and the shorter (47 kDa) fragment. Still, more significant inhibition was observed in modified, cancer-specific shorter forms. Reaching IC_50_ relative activity at cmpd No. 3, 15 µM and cmpd No. 9, 17 µM). In contrast, more significant differences were observed between the inhibitions of native and modified PFK-L enzymes. At lower concentrations, almost no deactivation of the native (85 kDa) activity was observed. However, more effective inhibition of the shorter (70 kDa) fragment of modified PFK-L was observed with cmpd No. 29, 30 (IC_50_ 8 µM) and cmpd No. 31, 32 (IC_50_ 9 µM). The data represents three independent measurements and are given as mean ±(SD) mean (n = 3).

Of the 33 compounds selected by screening, cmpds No. 3, 9, 23, 29, 30, 31, and 32 (marked in red in Fig 3) exhibited the ability to strongly suppress lactate or superoxide formation in at least one tumorigenic cell line (Table 1).

**Table 1:** A list of small-molecule inhibitors reducing human PFK1 iso-enzyme activities.

Compound No.	IUPAC name
3	2-[(5-benzo[1,3]dioxol-5-yl-1,3,4-oxadiazol-2-yl)sulfanyl]-N-isoxazol-3-yl-acetamide
9	3-methoxy-6-(3-{1-[(5-methyl-1,2,4-oxadiazol-3-yl)methyl]-1H-pyrazol-3-yl}phenyl)pyridazine
23	N-[5-(methanesulfonamido)-1,3,4-thiadiazol-2-yl]-6,7,8,9-tetrahydro-5H-carbazole-3-carboxamide
29	3-(4-chlorophenyl)sulfonyl-N-(5-isoxazol-5-yl-1,3,4-oxadiazol-2-yl)propenamide
30	4-[3-(5-amino-1,3,4-oxadiazol-2-yl)isoxazol-5-yl]phenol
31	(2R)-N-(5-isoxazol-5-yl-1,3,4-oxadiazol-2-yl)-2,3-dihydro-1,4-benzodioxine-2-carboxamide
32	3-(benzenesulfonyl)-N-(5-isoxazol-5-yl-1,3,4-oxadiazol-2-yl)propenamide

S1 Table and Text lists the inhibitors’ assigned numbers, structures, molecular weights, and supplier codes.

However, campuses No. 3 and 9 strongly inhibited unwanted metabolic activities in all tested tumorigenic cells. Similarly, cmpd No. 30 could also be considered a strong inhibitor, as a slightly lower level of SOX suppression was only observed in COLO 829 cells.

### Half maximal inhibitory concentrations (IC_50_) of selected compounds

To evaluate the ability of selected compounds to suppress the activities of the recombinant human native and modified PFK-M and PFK-L enzymes, they were constructed in yeast *S. cerevisiae* as a host organism (S1 Text), partially purified, and their activities assayed (S2 Text).

Among the inhibitors that preferentially bind to the PFK-P/PFK-M type ATP site, two cmpds (No. 3 and 9) showed potent inhibitions of the highly active modified sfPFK-M enzymes with half maximal inhibitory concentrations (IC_50_ values) of approximately 15 µM and 17 µM, respectively. Although cmpd No. 3 also significantly reduced the activity of the native nPFK-M enzyme (IC_50_, 18 µM), not as potent as inhibition of the native enzyme was observed by cmpd No. 9 (IC_50_, 41 µM).

Compounds targeting PFK-L-type ATP-binding sites also successfully inhibited the recombinant sfPFK-L enzyme. Inhibitors No. 29 and 30 showed potent inhibition of the highly active sfPFK-L enzyme (IC_50_, 8 µM each). Similar half-maximal inhibitory concentrations were observed with cmpds No. 31 and 32 (IC_50_, 9 µM each). The weakest inhibition was observed with cmpd No. 23 (IC_50_, 27 µM). However, all five inhibitors tested showed significantly weaker inhibitions of the native nPFK-L enzymes, ranging from IC_50_ values of 47–54 µM. Interestingly, compounds targeting the PFK-L type binding site inhibited the modifier sfPFK-L enzyme strongly concerning the native nPFK-L enzyme compared to the PFK-M type compounds (Fig 3).

Consequently, the individual inhibitors might act differently by preventing lactate and/or SOX formation in different tumorigenic cell lines. Some additional preclinical tests were performed to obtain more information on the efficiency of selected small molecule inhibitors for lactate/SOX suppression in cancer cells. Two compounds were selected for testing: cmpd No. 9 proved to be the most effective among compounds that preferentially dock to the ATP-binding type PFK-P/PFK-M, and cmpd No. 30 proved to be the most effective compound for lactate/SOX reduction among compounds that dock to the PFK-L isoenzyme.

#### A computational model of human PFK1 binding sites for two selected compounds.

**Fig 4:**
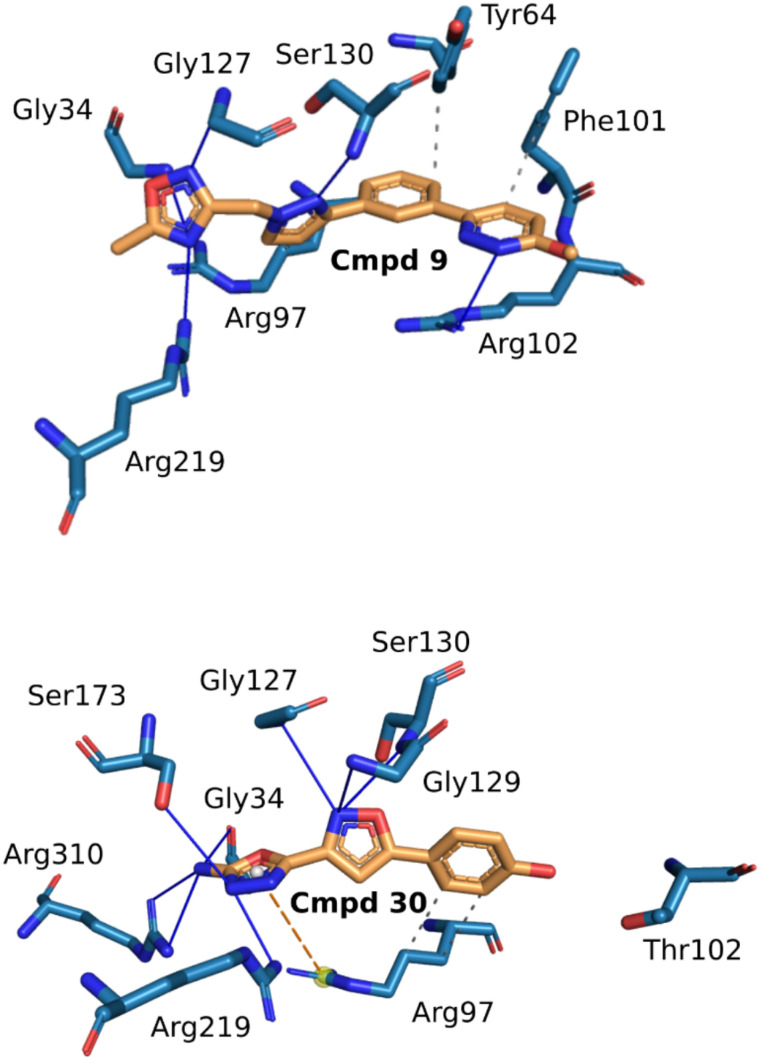
Computational models of catalytic binding sites of two PFK1 iso-enzymes as a docking target for small-molecule cmpd. (Above) shows a computer model of the amino acid residues of the PFK-P/PFK-M ATP binding sites with cmpd No. 9 as the ligand. (Below) shows a modified PFK-L model in which arginine was replaced by threonine and cmpd No. 30 as the ligand. A supercomputer used both models to virtual screen a ZINC Drugs NOW database.

The cmpd No. 9 interacts with the PFK-P/PFK-M binding site through a combination of hydrophobic interactions with Tyr64, Arg97, and Phe101 and multiple hydrogen bonds involving residues such as Gly34, Arg102, Gly127, Ser130, and Arg219 (Fig 4, above). The ligand cmpd No. 30 forms a stable complex with the PFK-L isoform through multiple non-covalent interactions: hydrophobic interactions with Arg97, extensive hydrogen bonds involving residues Gly34, Gly127, Gly129, Ser130, Ser173, Arg219, and Arg310, and a significant π-cation interaction with Arg97 (Fig 4, below). These interactions jointly stabilize both ligands within the protein binding site and contribute to the overall stability of the ligand-protein complexes.

#### Dose-dependent inhibition of lactate formation in different tumorigenic cell lines.

The lactate formation, growth yield, and survival under normal and inhibitory conditions of four tumorigenic cell lines were investigated. Initially, dose-dependent effects of selected inhibitors (cmpds No. 9 and 30) to suppress lactate formation were tested. Cells were grown in the presence of vehicles or inhibitors at different concentrations (20, 40, 60, and 80 µM), and lactate levels in the supernatant were determined sequentially.

**Fig 5:**
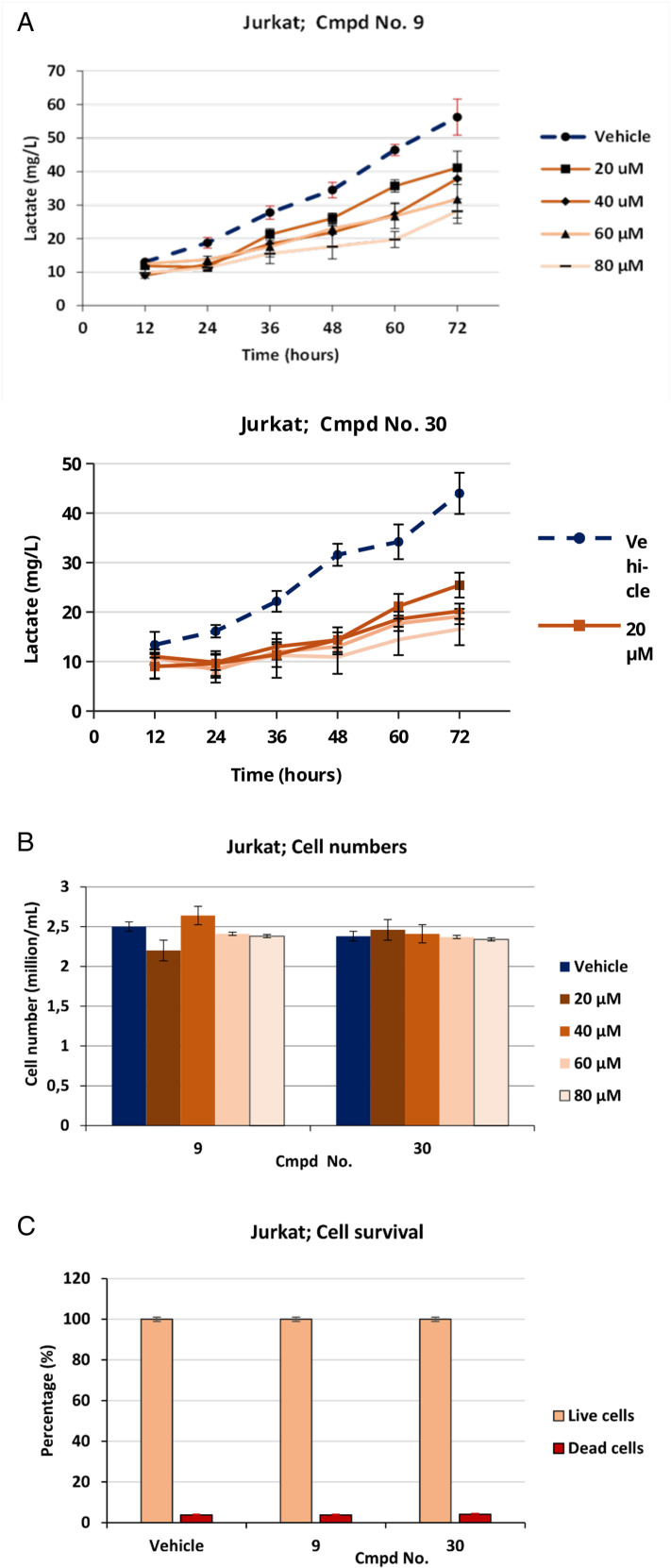
Dose-dependent reduction of lactate formation by Jurkat cells. Dose-dependent effects of cmpds No. 9 and 30 on lactate suppression were observed by the tumorigenic Jurkat cells. 5A. Simultaneously, no significant cytostatic (cells counted after 72 hours of incubation). 5B, and no cytotoxic effects (percentage of dead cells) after 72 hours of incubation). 5C was observed under dose-dependent conditions. Although initially (up to 24 hours), no reduced inhibitory effect of both compounds can be observed, the steadily increasing lactate formation in later incubation phases indicated an instability of the compounds. The data represents three independent measurements and are given mean ± SD (n = 3).

We observed that **Jurkat cells** in the presence of a vehicle started secreting lactate immediately after the start of incubation and continued to produce lactate until the end of the experiment at 72 hours. In contrast, cells treated with cmpds No. 9 and 30 produced no or only small amounts of lactate in the first 24 hours, regardless of the concentration of inhibitors in the medium. Still, they began to produce lactate in a dose-dependent manner as the incubation progressed. A somewhat stronger suppression of lactate formation was observed in the cells treated with cmpd No. 30 (Fig 5A).

To investigate possible cytostatic effects of the inhibitors, the total cell number of Jurkat cells treated with vehicles of different concentrations of inhibitors was determined immediately after incubation. No significant differences in the total number of cells were observed, regardless of the vehicle or the different concentrations of inhibitors in the media (Fig 5B).

The potential cytotoxicity of the inhibitors was also assessed. At the end of the incubation, the average percentage of dead cells was determined in the presence of a vehicle and a 80 µM concentration of each inhibitor. No significant adverse effects on Jurkat cell survival were observed in the presence of cmpds No. 9 (3.74 ± 0.3% dead cells) and No. 30 (4.04 ± 0.4% dead cells), which corresponds to the number of dead cells in the medium with vehicle (3.76 ± 0.35%) (Fig 5C).

A dose-dependent reduction in lactate accumulation was again observed when Caco-2 cells were tested with cmpds No. 9 and 30. However, in the medium with different concentrations of cmpd No. 9, lactate excretion was significantly reduced until about 24 hours after growth, followed by a dose-dependent increase in lactate formation in the late hours. Similar results were obtained in the medium with cmpd No. 30. In the media with vehicles or both inhibitors, a similar total number of cells was determined at the end of incubation, indicating that the inhibitors had no adverse effects on cell growth (S4 Fig). No cytotoxic effects of the inhibitors were observed either (S5 Fig). The average percentage of dead cells in control and the presence of the different concentrations of inhibitors was as follows: Vehicle (3.84 ± 0.54%), and No, 9 (3.33 ± 0.14%), and cmpd No. 30 (4.32 ± 0.51%) (S6 Fig).

In general, similar results of suppression of lactate formation were also obtained with **COLO 829 cells**. Apparent dose-dependent effects of suppressed lactate formation were observed with cmpd No. 9. Simultaneously, cmpd No. 30 proved slightly less efficient, as 80 µM reduced lactate formation by only about half. Again, both inhibitors were more effective only in the early phase of incubation (S7 Fig). No significant differences in the total cell number compared to the control were observed due to the different concentrations of the inhibitors in the medium (S8 Fig). The average percentage of dead COLO 829 cells in the presence of a vehicle and the presence of inhibitors was as follows: Vehicle (4.4 ± 0.46%), cmpd No. 9 (3.65 ± 0.78%) and cmpd 30 (4.27 ± 0.49) (S9 Fig).

Although the dose-dependent effect of the two inhibitors tested was also observed in the **MDA-MB-231 cells**, it was again only fully evident in the early phases of inoculation, while the weaker inhibition was more evident in the late hours of growth. In the early stages of growth, potent inhibition was observed by all concentrations, especially by cmpd No. 30. However, after about 24 hours, lactate accumulation resumed, initially at the lowest concentration (20 µM) and then gradually decreased in a dose-dependent manner (S10 Fig). No significant differences in total cell number were observed due to the different concentrations of inhibitors in the medium (S11 Fig). The average percentage of dead MDA-MB-231 cells in the control and the presence of the inhibitors was as follows: Vehicle (4.28 ± 0.78%), cmpd No. 9 (6.59 ± 0.29%), and cmpd No. 30 (3.4 ± 0.21%) (S12 Fig).

#### Sequential re-submission of inhibitors at low concentrations.

**Suppressing lactate generation:** In all dose-dependent tests, a gradual decrease in the inhibitory effect of the two compounds was observed after a certain period. The inhibitory potency appeared to disappear more rapidly at the lower concentrations, suggesting degradation of the compound in the medium or by cell metabolism. Therefore, the efficacy of selected inhibitors was evaluated by sequentially re-adding low concentrations at specific time points. In the experiment, the inhibitors were first added to the medium at lower concentrations at the start of inoculation and then added again at 24-hour intervals.

**Fig 6:**
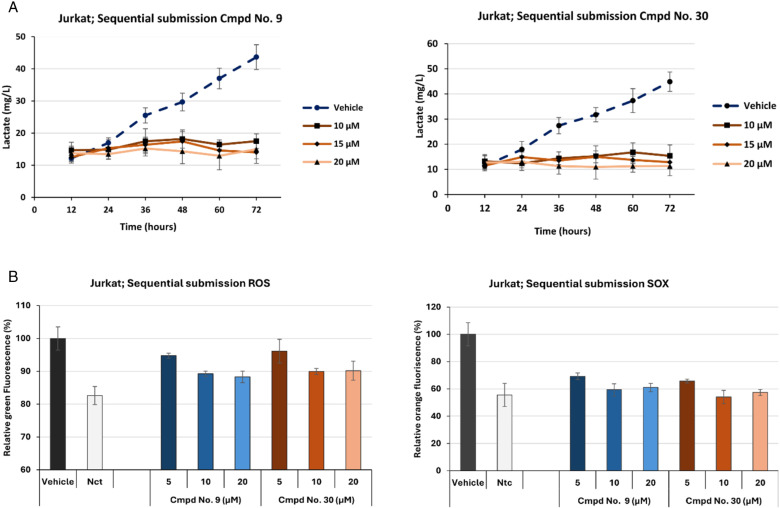
(a) Lactate suppression by sequential re-insertion of cmpd at low concentration in Jurkat cells. Lactate excretion in tumorigenic Jurkat cells remained effectively reduced when the cmpd No. 9 and 30) were added periodically every 24 hours at low concentrations (10, 15, and 20 µM). At 72 hours of incubation, the values of statistically significant differences between the vehicle and the cells treated with 10 µM of No. 9 and No. 30 were P < 0.0005, respectively. Data represents three independent measurements expressed as mean ±SD (n = 3). (b) Superoxide (SOX) and (ROS) suppression by sequential re-insertion of cmpds at low concentrations in Jurkat cells. Suppressed ROS and SOX formation was observed in Jurkat cells when cmpds No. 9 or 30 were sequentially added to the medium at low concentrations (5, 10, and 15 µM). Although some suppression of SOX formation was observed at 5 µM of both camps, significant differences were observed between the vehicle and cells treated with both cmpds (10 and 15 µM). The dose-dependent effects of the different concentrations of cmpds No. 9 and 30 were also examined regarding the suppression of ROS formation. However, only SOX formation in Jurkat cells appeared more potently suppressed. However, at 72 hours of inoculation, the cmpds tested for suppression reactive oxygen species (ROS) proved less efficient in reaching the P values of < 0.1, compared to the vehicle. Under identical growth conditions, the statistically significant values of SOX detected by 10 µM concentrations were P < 0.005 for No. 9 and P < 0.005 for No. 30, compared to the vehicle. The data are representative of three independent measurements and are given as mean ±SD (n = 3).

When lactate accumulation in **Jurkat cells** was monitored, convincing lactate suppression was observed with both inhibitors tested, even at the lowest inhibitor concentrations (10 µM). No significant increase in lactate concentration in the medium was observed within 72 hours after incubation (Fig 6A).

No cytotoxic effects were observed in **Jurkat cells** after 72 hours from the sequentially added inhibitors. The following average percentages of dead cells were observed at the end of fermentation in the cells in the presence of a vehicle (3.21 ± 0.28%), and the cells were sequentially treated with 10 µ L cmpd No. 9 (3.24 ± 0.25%) and cmpd No. 30 (3.64 ± 0.23%).

In both inhibitors tested, sustained inhibition of lactate formation was observed in **Caco-2 cells**. Almost no dose-dependent effect of cmpd No. 9 and cmpd No. 30 was observed at all concentrations tested. This indicates that effective suppression of lactate formation could be prevented at the lowest 10 µM concentrations (S13 Fig).

No significant cytotoxic effects of the inhibitors were observed after 72 hours of incubation, although different concentrations of inhibitors were added sequentially. The following average percentages of dead cells were observed in the medium without added inhibitors (4.45 ± 0,41%) and with the cells sequentially treated with 10 µM cmpd No. 9 (4.03 ± 0.32%), and cmpd No. 30 (4.24 ± 0.38%).

In **COLO 829 cells**, slightly weaker lactate formation was observed at 10 µM concentrations in the presence of both inhibitors, while extensive suppression was observed at 15 and 20 µM concentrations (S14 Fig).

After 72 hours of incubation, no significant cytotoxic effect of the inhibitors could be detected, although different concentrations of inhibitors were added sequentially. The following average percentages of dead cells were observed in the medium without adder inhibitors (2.44 ± 0.25%) and with the cells sequentially treated with 10 µM cmpd No. 9 (2.84 ± 0.19%) and cmpd No. 30 (2.42 ± 0.21%).

Similar results of suppressed lactate formation with inhibitors were obtained in **MDA-MB-231** cells (S15 Fig). No significant cytotoxic effect of the inhibitors could be detected after 72 hours. The following average percentages of dead cells were observed in the medium without added inhibitors (2.52 ± 0.356%) and with the cells sequentially treated with 10 µM inhibitor No. 9 (2.35 ± 0.09%) and inhibitor No. 30 (2.32 ± 0.21%).

**Suppressing ROS/SOX generation:** Selected PFK1 inhibitors (cmpds No. 9 and 30), thought to reduce cytosolic NADH, primarily prevent SOX formation but not other oxygen stress reagents. Accordingly, the dose-dependent effect of the two inhibitors in reducing ROS formation was lower. While the 5 µM concentration showed an approximately 50% reduction, the 10 and 20 µM concentrations reduced ROS formation by approximately 65%. The maximum decrease in ROS formation appeared to be achieved at a concentration of about 10 µM, characteristic of all tumorigenic cell lines tested. The following statistical significance differences between the vehicle and the cells treated with inhibitors were obtained: Figs 6B and S16–S18).

In terms of reduction in ROS formation, more significant suppression of SOX levels was observed by the two selected PFK1 inhibitors in all tumorigenic cell lines. A decrease in SOX concentration of about 70% was observed at a 5 µM concentration of both inhibitors, while at a concentration of 10 µM, virtually no SOX was present.

It is worth noting that in COLO 829 cells treated with both inhibitors, the most significant statistical differences were observed in the suppression of SOX generations compared to the other tumorigenic cells tested. However, the evaluation of lactate suppression by both inhibitors showed the least significant differences in COLO 829 cells compared to other tumorigenic cells tested.

### Effects of compounds on respiration and glycolytic rates in treated and untreated tumorigenic Jurkat cells

The inhibitors’ efficiency was also tested by measuring respiratory flow, maximal respiratory rate, glycolytic rate, and glycolytic capacity in Jurkat cells. Bioenergetic studies were performed using the Seahorse XFp assay (Agilent Technology), which allows non-destructive measurements of the oxygen consumption rate (OCR) and extracellular acidification rate (ECAR) of the cells tested.

First, basal glycolytic respirations and basal glycolytic fluxes were determined by measuring the OCR and ECAR values respectively, in the untreated Jurkat cells (vehicle) and the cells treated with cmpd No. 9 or No. 30.

Lower respiration levels were detected in the cells treated by cmpds No. 9 and 30 compared to the untreated cells. Similarly, measurements of basal glycolytic rates in the media with added cmpds were observed. However, stronger statistical significances were obtained from extracellular acidification rate measurements compared to respiration.

After 20 minutes of incubation, immediately after the samples for respiration and glycolysis have been taken, Oligomycin A, an inhibitor of mitochondrial ATP synthase, and FCCP, a protonophore causing a breakdown of the mitochondrial inner membrane that disrupts hydrogen ions transport, were added to the medium. Dysfunctional mitochondrial oxidative phosphorylation is supposed to induce glycolytic stress, which enables the determination of maximal respiration and glycolytic capacity.

**Fig 7:**
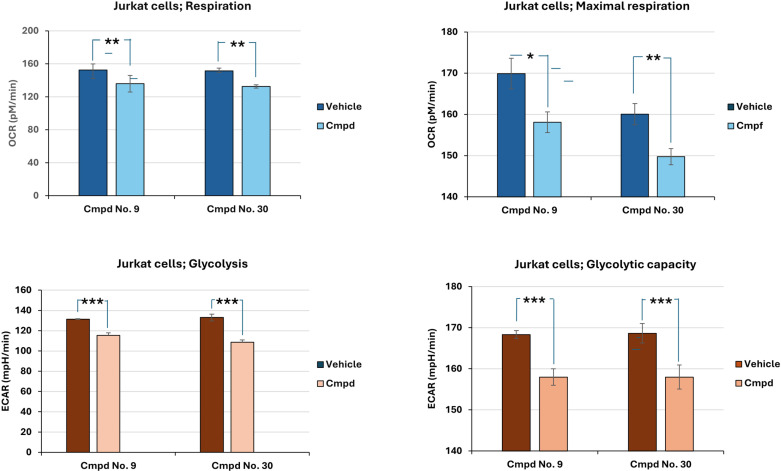
The influence of selected PFK1 compounds on glycolytic and respiratory parameters in Jurkat cells. Respiratory fluxes, maximal respiration rates, glycolytic rates, and glycolytic capacities were measured in Jurkat cells in the presence and absence of selected cmpd. Oxygen consumption rate (OCR) and extracellular acidification rate (ECAR), measured in the first 20 minutes of the experiment, were significantly higher in the untreated cells (vehicle) than in the treated cells. For cmpd No. 9, the statistical significances between untreated and treated cells were P** < 0.01 for respiration and P*** < 0.005 for glycolytic rate, while for cmpd No. 30, significances of P** < 0.01 for respiration and P*** < 0.005 for glycolytic rate, were determined. After 20 minutes, oligomycin A and FCCP were added, which made it possible to evaluate the maximal respiration and glycolytic capacities. In Jurkat cells treated with cmpd No. 9, the highest values of < P* 0,05 were obtained for maximal respiration and P*** value <0.005 for glycolytic capacity, compared to the vehicle while in the cells treated with cmpd No. 30, the values for maximal respiration were P** < 0.01 and for glycolytic capacity P*** < 0.005, compared to the vehicle. Data represents three independent measurements expressed as mean ±SD (n = 3).

Samples collected 6.5 minutes after the first sampling showed a more substantial increase of respiration and acidification in the cells treated with cmpd No. 9 or No. 30 compared to the vehicle. However, maximal oxygen consumption levels and glycolytic capacities in the treated cells remained lower than those of the untreated cells (Fig 7). The graphics of the analysis in time of the experiments are shown in the Supplementary materials (S19 Fig).

It is worth noting that tumorigenic Jurkat calls treated by cmpds can reduce oxygen consumption rate and (ECAR) exocellular acidification rate and inhibit cancer-specific PFK1 activities that subsequently subsidize deleterious uncontrolled energy metabolize in treated tumorigenic cells.

### Apoptotic assays in a co-culture of tumorigenic Jutkat cells and activated T-cells

**Fig 8:**
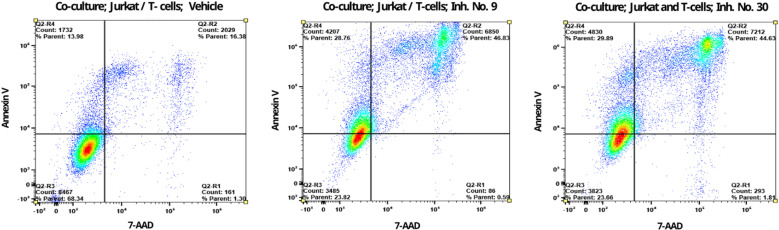
In the co-culture of tumorigenic Jurkat cells and activated T-cells, apoptosis is enhanced in the presence of PFK1 cmpd. It is assumed that extracellular lactate in tumors triggers an acidosis that prevents the normal functioning of immune cells. Since PFK1 cmpds are thought to suppress lactate formation, acidosis may not be formed, so fully functional T cells could defeat cancer cells by apoptosis. Accordingly, increased late apoptosis was expected in a co-culture of activated T cells and tumorigenic Jurkat cells treated with PFK1 cmpds. In fact, after 18 hours of co-culture incubation with untreated cells (vehicle), only 18% (Q2-R2) of the cells were detected at the late apoptosis stage using a flow cytometer. However, in the cells treated with 10 µM cmpd No. 9 under identical environmental conditions, significantly higher proportions of cells were observed in the late apoptosis stage (Q2-R2). No. 9 (46.83%) or 10 µM of cmpd No. 30 (44.63%).

Acidifying the tumor matrix by extracellular lactate accumulation may be an essential factor in evading destruction by the immune system, as it is thought to interfere with the standard functionality of immune cells in destroying cancer cells [19]. It is hypothesized that reduced formation and transmembrane transport of lactate could prevent a drop in pH in the tumor microenvironment while restoring regular immune cell activity to induce apoptotic death of cancer cells. To test this, tumorigenic Jurkat cells were incubated with activated human T lymphocytes in a co-culture with treated or untreated cells. A medium without bicarbonate or other buffers was used in the experiments to maintain acidosis. After 18 hours of co-culture incubation, limited and extensive apoptotic cell death was assessed by detecting early-stage (AnnexinV+/7-AAD-)(Q2-R4) and late-stage (AnnexinV+/7-AAD+)(Q2-R2) apoptosis. The flow cytometer results showed a significantly increased percentage of late apoptosis in cells treated with cmpds No. 9 or 30 at 46.83% (Fig 8B) and 44.63% (Fig 8C), respectively. In comparison, only 16.38% of late apoptotic cells were observed in untreated Jurkat cells in a co-culture with activated T cells (vehicle) (Fig 8A). These observations clearly show for the first time that this class of PFK1 inhibitors can accelerate apoptosis and result in a faster progression of late apoptosis than in untreated cells.

## Discussion

The drugs described so far that reduce cancer-specific glycolytic fluxes are cisplatin, and etoposide [30]. clotrimazole [31], and taxol [32]. Taxol induces PFK detachment from the cytoskeleton and has been reported to affect PFK1 activity by reducing gene expression. However, these agents act not only by reducing the transcription of genes but also by other glycolytic genes. For example, cisplatin, used for the clinical treatment of testicular, ovarian, head and neck, non-small cell lung cancer, and recurrent lymphoma, impairs transcription and replication, promoting apoptosis [33]. However, glycolytic flux in eukaryotic organisms is tightly controlled by allosteric enzymes, which maintain their regulation by feedback inhibition despite the increased activities of intermediate enzymes. Therefore, specific intervention in an irreversible, rate-limiting step of glycolysis may be more effective in reducing dysregulated glycolytic flux.

In the past, numerous attempts have been made to target enzymes to treat impaired energy metabolism, which causes lactate formation and increased ROS levels in cancer cells. The following enzymes have been most frequently targeted: Hexokinase, 6-phosphofructo-2-kinase, lactate dehydrogenase, mono-carboxylase, and pyruvate dehydrogenase [34], but not 6-phosphofructo-1-kinase.

Interestingly, inhibition of lactate dehydrogenase (LDHA) by the small molecule inhibitor FX11 led to a decrease in lactate and ATP concentration in the tumors but simultaneously caused a significant increase in ROS concentration, which triggered oxidative stress and eventually cell death [35].

Similar effects, namely the suppression of lactate formation and the excessive formation of ROS, have been observed with the cancer drug dichloroacetate (DCA). It could be used to treat hereditary lactic acidosis. DCA indirectly stimulates the activity of pyruvate dehydrogenase and diverts pyruvate from lactate formation to mitochondrial catabolism [36]. Although some tests suggest that DCA is effective and well tolerated by patients with glioblastoma, it should be emphasized that the dysregulated glycolytic flux leaves cytosolic NADH abundance unchanged, so that ROS formation is enhanced by OXPHOS, causing oxidative stress [37]. The resulting mitochondrial overload impairs the efficiency of the antioxidant system, which cannot cope with the excessive amount of ROS. Recently, it has been shown that the lack of well-equipped machinery capable of coping with sustained oxidative phosphorylation leads to damage to the nervous system and, thus, to peripheral neuropathy [38].

Much research has been done in the past on 6-phosphofructo-2-kinase/fructose-2,6-bisphosphatase (PFKB3), which converts fructose-6-phosphate (F6P) to fructose-2,6-bisphosphate (F2,6BP), a potent activator of 6-phosphofructo-1-kinase (PFK1) [39]. As a vital regulator of glycolysis, numerous studies suggest that PFKB3 is indirectly associated with many aspects of cancer, including carcinogenesis and cancer cell proliferation. F2,6BP not only acts as a potent PFK1 activator, enabling increased glycolytic flux but also activates cyclin-dependent kinases (Cdks) and then stimulates Cdk-mediated phosphorylation of the Cip/Kip protein p27. Mediated p27 phosphorylation, in turn, leads to a decrease in p27 levels due to ubiquitination and proteasomal degradation by Cdk1. Since p27 is a potent suppressor of the G1/S transition and activator of apoptosis, inhibition of PFKB3 and the concomitant decrease in F2,6BP levels could also prevent p27 degradation and, consequently, cell cycle arrest [40]. Indeed, the compound 3PO, a potent inhibitor of PFKB3, was found to cause a slowdown in the G2-M phase preceded by a reduction in F2,6BP levels [41]. Although 3PO and its derivatives were initially shown to inhibit lactate formation, the more important therapeutic benefit of these inhibitors in cancer treatment appears to be their specific cytotoxicity. However, the induced cell cycle arrest makes the compound unsuitable for combination with immunotherapy.

Rather than reducing PFKB3 activities, which leads to a reduction in F2,6BP levels, partial inhibition of PFK1 activities by small molecule inhibitors appears to be the more straightforward mechanism to control dysregulated glycolytic flux in cancer cells.

However, no specific inhibitors were tested for the iso-enzymes of 6-phosphofructo-1-kinase (PFK1), which are irreversible key enzymes of glycolysis that could act as a key factor in impaired metabolism. We were the first to report that post-translationally modified PFK1 enzymes were present in the cancer cells. While the 47 kDa fragment was predominant in the modified PFK-M isoform [2], the 70 kDa form was characteristic of the modified PFK-L isoform [12]. Interestingly, systemic deletions and point mutations in the C-terminus of mammalian PFK1 cells have identified two amino acid residues (Leu-767 and Glu-768) that allow allosteric inhibition by MgATP. These amino acids are located at the very end of the C-terminus of human PFK1. Leu-767 and Glu-768 are the only two residues of the C-terminus that are conserved in all mammalian isoenzymes. Furthermore, their deletion leads to a disruption of inhibition by citrate and MgATP and, thus, to a dysregulation of PFK1. These results suggest that the truncation length is irrelevant to deactivating the allosteric negative control [42]. Indeed, for IC_50_ determination, similar results of lactate suppression were observed with purified shorter PFK-M enzymes (45 kDa) and purified shorter PFK-L enzymes (70 kDa).

Highly active, cancer-specific shorter PFK1 fragments were previously overlooked mainly because of their extreme instability under in vitro conditions. Rapid deactivation was observed during enzyme activity measurements. It appears that cleavage of the C-terminus of the holoenzyme is known to stabilize the tetrameric structure of the native enzyme, making the enzyme more susceptible to dissociation. It looks likely that the shorter fragments, which lack most of the C-terminus, can only assemble in dimeric forms [13]. The inactivation of PFK1 enzymes by dissociation at low protein concentrations has been previously reported and well characterized for the rat liver enzyme [43,44].

Corrupted control of glycolytic flux at the level of PFK1 in cancer cells was indirectly confirmed by other authors. Analysis of two rapidly growing cell types, human HeLa cells, and rodent AS-30D cells, showed a lack of allosteric control at the level of PFK1, which was also confirmed by kinetic analyses of the enzyme [45]. In experiments with PC3 cells, prostate cancer-derived cells, an overexpressed short-form, citrate-resistant PFK1 was detected. In addition, the citrate-resistant cells accumulated more mitochondrial ROS than the control cells [46]. Shorter cancer-specific liver-type PFK-L were described in several tumorigenic cells with overexpressed Tap73, a structural homolog of the tumor suppressor p53. By Western analysis, only shorter 70 kDa PFK-L fragments were detected during growth under enhanced proliferation conditions to promote the Warburg effect [47].

However, not only cancer cells, but also rapidly proliferating normal cells require increased nutrient uptake and catabolism to support cell growth. Therefore, increased glycolytic flux typically occurs in normal human cells. In normal, rapidly proliferating stem cells, different types of primary anabolic metabolism can be found that play a crucial role in whether a cell proliferates, differentiates, or remains quiescent [48]. In proliferating hematopoietic cells, for example, anabolic glycolysis is activated, which enables the simultaneous formation of lactate and ROS. In mesenchymal stem cells differentiating into adipocytes, high OXPHOS and ROS formation were found, whereas, in mesenchymal cells differentiating into osteoblasts and chondroblasts, ROS formation was blocked. In contrast, in differentiated embryonic cells, a reduced glycolytic flux prevents lactate formation, while ROS formation is possible [49].

It should be emphasized that the proliferative metabolism of normal cells is tightly controlled by growth and transcription factors and other signaling mechanisms that enable the cells to differentiate into other cell types. Metabolic activities also change during the different stages of the cell cycle. A ROS-enriched environment, as found in some forms of proliferating normal cells, can trigger mutations that can lead to carcinogenesis [50]. It is generally believed that dysregulated glycolysis is an early event in oncogenesis that is a direct consequence of the first mutations [33]. For example, KRAS in pancreatic cancer and BRAF in melanoma occur before cell invasion in benign and early lesions [51,52].

## Conclusion

Selected small molecule inhibitors shown to diminish cancer-specific modified forms of PFK1 may be a suitable way of reducing dysregulated glycolytic flux in cancer cells. The devastating metabolic flux typical of cancer cells can thus be controlled and reach that of non-proliferating normal cells. Most importantly, the redox balance is restored, eliminating the need to re-oxidize excessive amounts of cytosolic NADH by the simultaneous lactate and superoxide formation.

Since elevated levels of ROS are thought to trigger neoplastic signaling and equally devastating mutations, modified PFK1 inhibition could thwart the development of several pre-cancerous hallmarks, such as genome instability and mutations, dysregulated cell metabolism, maintenance of proliferation signaling, evasion of growth suppressors, facilitation of replicative immortality, and resistance to cell death.

**Fig 9:**
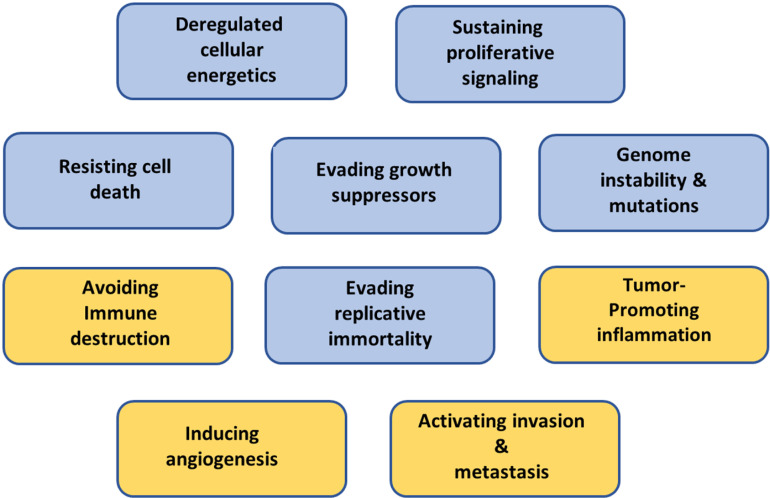
Schematic presentation of lactate and superoxide functions in cancer development. The schematic shows the involvement of lactate and superoxide in the development of solid tumors with specific cancer characteristics. While extracellularly elevated lactate levels trigger acidosis in the tumor matrix, which leads to immune response, contributes to chronic inflammation, stimulates angiogenesis, and promotes metastasis formation, superoxide is considered a precursor of hydroxyl radicals, the most potent endogenous mutagen. The involvement of lactate (yellow) and superoxide (blue) in carcinogenesis is illustrated in the scheme previously published in “Hallmarks of Cancer: The Next Generation” [1].

At the same time, preventing lactate formation could preclude mechanisms involved in developing other cancer hallmarks, such as triggering angiogenesis, immune escape, tumor-promoting inflammation, and metastasis (Fig 9).

The accumulation of lactate and ROS is the key factor in developing solid tumors. These arguments support the hypothesis that cancer-specific, highly active modified PFK1s are the “key players” in cancer development and progression. Therefore, reduced PFK1 activity in cancer cells could prevent early development in the pre-cancerous stage and progression in the later stages of invasive cancers.

## Supporting information

S1 Text PFK1 Cloning, expression, purification, and measurements. (PDF)

S2 Text PFK1 Enzymatic assays. (PDF)

S1 Table and Text Small-molecule inhibitors. (PDF)

S1 Fig Preliminary screening of selected compounds in colorectal adenocarcinoma Caco-2 cells. (PDF)

S2 Fig Preliminary screening of selected compounds in melanoma COLO 829 cells. (PDF)

S3 Fig Preliminary screening of selected compounds in breast gland adenocarcinoma MDA-MD-231 cells. (PDF)

S4 Fig Dose-dependent inhibition of lactate formation in Caco-2 cells. (PDF)

S5 Fig Dose-dependent inhibition of lactate formation in Caco-2 cells – cytostatic effect. (PDF)

S6 Fig Dose-dependent inhibition of lactate formation in Caco-2 cells – cytotoxic effect. (PDF)

S7 Fig Dose-dependent inhibition of lactate formation in COLO-829 cells. (PDF)

S8 Fig Dose-dependent inhibition of lactate formation in COLO-2 cells – cytostatic effect. (PDF)

S9 Fig Dose-dependent inhibition of lactate formation in COLO cells – cytotoxic effect. (PDF)

S10 Fig Dose-dependent inhibition of lactate formation in MDA-MB-231. (PDF)

S11 Fig Dose-dependent inhibition of lactate formation in MDA-MB-231 cells – cytostatic effect. (PDF)

S12 Fig Dose-dependent inhibition of lactate formation in MDA-MB-231 cells – cytotoxic effect. (PDF)

S13 Fig Lactate suppression by sequential re-insertion of inhibitors at low concentration in Caco-2 cells. (PDF)

S14 Fig Lactate suppression by sequential re-insertion of inhibitors at low concentration in COLO 829 cells. (PDF)

S15 Fig Lactate suppression by sequential re-insertion of inhibitors at low concentration in MDA-MB-231 cells. (PDF)

S16 Fig Superoxide (SOX) and reactive oxygen species (ROS) suppression by sequential re-insertion of inhibitors at low concentrations in Caco-2 cells. (PDF)

S17 Fig Superoxide (SOX) and reactive oxygen species (ROS) suppression by sequential re-insertion of inhibitors at low concentrations in COLO 829 cells. (PDF)

S18 Fig Superoxide (SOX) and reactive oxygen species (ROS) suppression by sequential re-insertion of inhibitors at low concentrations in MDA-MB-231 cells. (PDF)

S19 Fig The graphs for OCR and ECAR calculations on Jutkat cells were analyzed in time. (PDF)

## Abbreviations

HK2: Hexokinase 2
nPFK1: native human 6-phosphofructo-1-kinase
sfPFK1: human short fragment 6-phosphofructo-1-kinase
n*PFK1*: gene encoding human nPFK1
sf*PFK1*: gene encoding human sfPFK1
*pfk*: gene encoding yeast 6-phosphofructo-1-kinase
PFKB3: 6-phosphofructo-2-kinase/fructose-2,6-bisphosphatase
F6P: fructose-6-phosphate
F2,6BP: fructose-2,6-bisphosphate
GAPDH: glyceraldehyde-3-phosphate dehydrogenase
LDHA: lactate dehydrogenase
Cdks: cyclin-dependent kinases
G2-M phase: DNA damage checkpoint
NADH: Nicotinamide adenine dinucleotide (reduced)
NAD^+^: Nicotinamide adenine dinucleotide (oxidized)
OXPHOS: Oxidative phosphorylation
SOX: Superoxide
ROS: Reactive oxygen species
cmpd: Compound
