# Supplementary material for: Small-molecule inhibitors of 6-phosphofructo-1-kinase simultaneously suppress lactate and superoxide generation in cancer cells
Source: PLoS One. 2025 May 21;20(5):e0321998. doi: 10.1371/journal.pone.0321998 (PMC12094722; doi:10.1371/journal.pone.0321998)
Supplement: S1 Text — (PDF) [file pone.0321998.s001.pdf]

S1 Text: PFK1 Cloning, expression, purification, and measurements.

Recombinant genes construction and transformation into pfk-null S.cerevisiae strain

The human genes encoding native n*PFKM* (UniProt P08237), n*PFKL* (UniProt P17858-1) muscle type PFK-M of 85.051 Da, and native liver type PFK-L of 84.917 Da were constructed as reported previously [1]. The identical method was used to build truncated human genes sf*PFKM* and sf*PFKL*, encoded short PFK-M of 45.920 Da [2] and short PFK-L of 69.942 Da fragments [3], respectively. Individual genes were inserted into *pfk*-null HD-114-8A yeast *S.cerevisiae*.

Purification of recombinant PFK1 proteins

Recombinant human PFK1 native and short fragments synthesized as yeast transformants were partially purified, as reported previously [2].

References:

1. Mumberg D, Müller R, Funk M. Yeast vectors for the controlled expression of heterologous proteins in different genetic backgrounds. Gene. 1995;156: 119–122. Available: http://www.ncbi.nlm.nih.gov/pubmed/77375042.

2. Andrejc D, Možir A, Legiša M. Effect of the cancer-specific shorter form of human 6-phosphofructo-1-kina on the metabolism of the yeast *Saccharomyces cerevisiae*. BMC Biotechnol. 2017;17: 1-13. doi:10.1186/s12896-017-0362-5.

3. Kristl A, Čamernik K, Avbelj Š, Legiša M. Another Form of Modified, Highly-Active 6-Phosphofructo-1-Kinase in Cancer Cells. Ann Hematol Oncol. 2021;8. Available: doi:10.26420/annhematoloncol.2021.1344
