# Supplementary material for: Small-molecule inhibitors of 6-phosphofructo-1-kinase simultaneously suppress lactate and superoxide generation in cancer cells
Source: PLoS One. 2025 May 21;20(5):e0321998. doi: 10.1371/journal.pone.0321998 (PMC12094722; doi:10.1371/journal.pone.0321998)
Supplement: S2 Text — (PDF) [file pone.0321998.s002.pdf]

S2 Text: PFK1 Enzymatic assays.

PFK1 activities were determined spectrophotometrically at 340 nm (Lambda 25 UV/VIS spectrophotometer, Perkin Elmer) as reported previously [1] using a coupled reaction system. Unless otherwise stated, in a final volume of 1 mL, the assay mixture contained 50 mM HEPES buffer (pH 7.8), 1 mM DTE, 100 mM KCl, 5 mM $MgCl_2$, 0.2 mM NADH, 0,025 to 2 mM F6P, 10% v/v polyethylene glycol (PEG 6000) [2], 0.9 U/mL aldolase (Sigma-Aldrich, Steinheim, Germany), 15 U/mL triosephosphate isomerase, and 15 U/mL glycerol-3-phosphate dehydrogenase (Sigma-Aldrich, Steinheim, Germany). Before use, the auxiliary enzymes were dialyzed overnight at 4ºC against 50 mM HEPES buffer (pH 7.8) containing 1 mM DTE, with one buffer change after 8h. The ATP concentration (0.5 mM) enabled optimal PFK1 activities but was below the limit to induce inhibition. The enzyme concentration used in the kinetic assay was 0.2 µg/mL. All the substrates (Fructo-6-phosphate (F6P) and ATP), auxiliary enzymes, NADH, and inhibitors dissolved in DMSO were first added to the PEG buffer for the enzymatic measurements. After thoroughly mixing the buffer with all ingredients, the enzymatic reactions started by inserting the purified recombinant enzymes. All presented kinetic data are averages obtained from at least three replicate measurements. Total protein concentrations of the samples were determined using a Bio-Rad protein assay (Bio-Rad, Hercules, Ca) with bovine y-globulin as a standard.

References:

1. Mumberg D, Müller R, Funk M. Yeast vectors for the controlled expression of heterologous proteins in different genetic backgrounds. Gene. 1995;156: 119–122. Available: http://www.ncbi.nlm.nih.gov/pubmed/77375042.
2. Aragon JJ, Sols A. Regulation of enzyme activities in the cell: Effect of enzyme concentration. Faseb J, 1991;5:29945-2950. doi: 10.1096/fasebj.5.14.1752361.
