# Supplementary material for: Small-molecule inhibitors of 6-phosphofructo-1-kinase simultaneously suppress lactate and superoxide generation in cancer cells
Source: PLoS One. 2025 May 21;20(5):e0321998. doi: 10.1371/journal.pone.0321998 (PMC12094722; doi:10.1371/journal.pone.0321998)
Supplement: S1 Table and Text — (PDF) [file pone.0321998.s003.pdf]

S1 Table and Text: Small-molecule inhibitors.

List of selected compounds

Compound No.	IUPAC name	Structure	MW	Supplier code
**1**	5-[[[(3R)-1-(2,3-dihydro-1,4-benzodioxin-6-yl)-5-oxo-pyrrolidine-3-arbonyl]amino]methyl]isoxazole-3	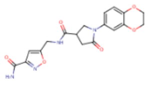	386,364	Z1407829627
**2**	N-[(1S)-tetralin-1-yl]-3-[3-(4H-1,2,4-triazol-3-yl)-1,2,4-oxadiazol-5-yl]propenamide	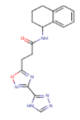	338,371	Z2241119275
**3**	2-[(5-benzo[1,3]dioxol-5-yl-1,3,4-oxadiazol-2-yl)sulfanyl]-N-isoxazol-3-yl-acetamide	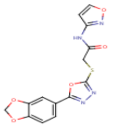	346,324	Z225001190
**4**	1-(2,3-dihydro-1,4-benzodioxine-6-sulfonyl)-N-(5-methyl-1,3,4-oxadiazol-2-yl)piperidine-4-carboxamide	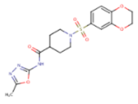	408,436	Z26691022
**5**	N-(5-methyl-1,3,4-oxadiazol-2-yl)-1-(2-naphthylsulfonyl)piperidine-4-carboxamide	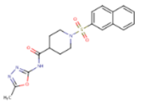	400,46	Z26691362
**6**	N-(5-methyl-1,3,4-oxadiazol-2-yl)-1-tetralin-2-ylsulfonyl-piperidine-4-carboxamide	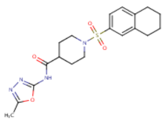	404,492	Z51747153
**7**	4-(1,3,4-oxadiazol-2-yl)phenyl 2,3-dimethylquinoxaline-6-carboxylate	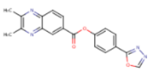	346,346	Z90863115
**8**	(5-methyl-1,3,4-oxadiazol-2-yl)methyl 3-phenyl-2,1-benzoxazole-5-carboxylate	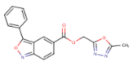	335,319	Z92739596

**9**	3-methoxy-6-(3-{1-[(5-methyl-1,2,4-oxadiazol-3-yl)methyl]-1H-pyrazol-3-yl}phenyl)pyridazine	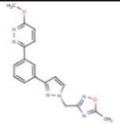	348,366	28988176
**10**	4-{[3-(1,3-benzodioxol-5-yl)-1,4,6,7-tetrahydro-5H-pyrazolo[4,3-c]pyridin-5-yl]methyl}-1H-pyrazole-3-carboxylic acid	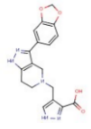	367,365	39527583
**11**	3-{[3-(3-fluorophenyl)-6,7-dihydroisoxazolo[4,5-c]pyridin-5(4H)-yl]methyl}-N-methyl-1,2,4-oxadiazole-5-carboxamide	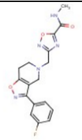	357,345	42443345
**12**	5-{[3-(1,3-benzodioxol-5-yl)-6,7-dihydroisoxazolo[4,5-c]pyridin-5(4H)-yl]methyl}-1,2,4-oxadiazole-3-carboxamide	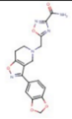	369,337	56880011
**13**	5-{[3-(1,3-benzodioxol-5-yl)-6,7-dihydroisoxazolo[4,5-c]pyridin-5(4H)-yl]methyl}-1,2,4-oxadiazole-3-carboxamide	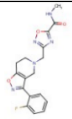	357,345	60395053
**14**	3-methyl-6-(3-{1-[(5-methyl-1,3,4-oxadiazol-2-yl)methyl]-1H-pyrazol-3-yl}phenyl)pyridazine	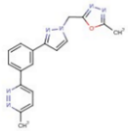	332,367	77231175
**15**	5-{[2-(6-methoxy-2-naphthyl)morpholin-4-yl]methyl}-1,2,4-oxadiazole-3-carboxamide	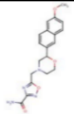	368,393	84820077
**16**	3-{[3-(1,3-benzodioxol-5-yl)-6,7-dihydroisoxazolo[4,5-c]pyridin-5(4H)-yl]methyl}-1,2,4-oxadiazole-5-carboxamide	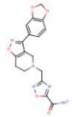	369,337	92386041
**17**	N-(2-hydroxy-5-methyl-phenyl)-4-(3-nitro-1,2,4-triazol-1-yl)butyramide	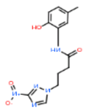	305,294	C163-0463
**18**	N-(4-sulfamoylphenyl)-4H-chromeno[3,4-d]isoxazole-8-sulfonamide	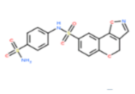	407,429	M030-0076

**21**	2-[(5-methyl-1,2,4-oxadiazol-3-yl)methylsulfonyl]-N-[(1R)-tetralin-1-yl]acetamide	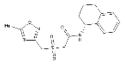	349,412	Z417063332
**22**	2,3-dimethyl-N-[3-(methylsulfonylmethyl)-1,2,4-oxadiazol-5-yl]-1H-indole-5-carboxamide	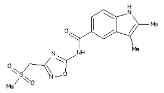	348,384	Z1230053398
**23**	N-[5-(methanesulfonamido)-1,3,4-thiadiazol-2-yl]-6,7,8,9-tetrahydro-5H-carbazole-3-carboxamide	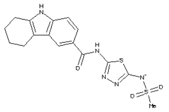	391,478	Z750400774
**24**	2-(5-methylisoxazol-3-yl)oxy-N-[3-(1-methyltetrazol-5-yl)phenyl]acetamide	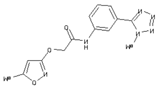	314,305	Z226594542
**25**	[3-(1,3,4-oxadiazol-2-yl)phenyl]	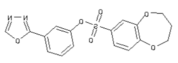	374,374	Z357948840
**26**	N-(5-methyl-1,3,4-oxadiazol-2-yl)-1-(p-tolylsulfonyl)piperidine-4-carboxamide	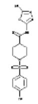	364,427	Z26690411
**27**	2-[3-(4H-1,2,4-triazol-3-ylsulfonyl)propyl]-1,3-benzoxazole	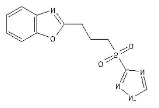	292,32	Z1385366083
**28**	N,4-dimethyl-N-[4-(1,3,4-oxadiazol-2-yl)benzyl]benzenesulfonamide	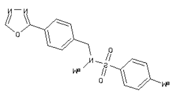	343,408	Z55670817
**29**	3-(4-chlorophenyl)sulfonyl-N-(5-isoxazol-5-yl-1,3,4-oxadiazol-2-yl)propenamide	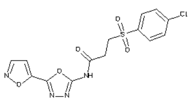	382,785	Amb1379074
**30**	4-[3-(5-amino-1,3,4-oxadiazol-2-yl)isoxazol-5-yl]phenol	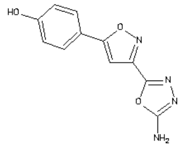	244,21	Amb6474776
**31**	(2R)-N-(5-isoxazol-5-yl-1,3,4-oxadiazol-2-yl)-2,3-dihydro-1,4-benzodioxine-2-carboxamide	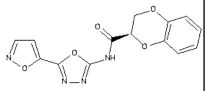	314,257	Amb7179940

**32**	3-(benzenesulfonyl)-N-(5-isoxazol-5-yl-1,3,4-oxadiazol-2-yl)propenamide	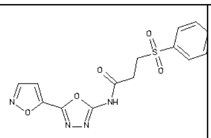	348,34	Amb1379063
**34**	N-(5-isoxazol-5-yl-1,3,4-oxadiazol-2-yl)-2-(2-naphthyl)acetamide	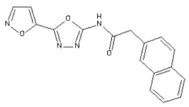	320,308	Amb1379145
**35**	2-(4-fluorophenyl)sulfonyl-N-(5-isoxazol-5-yl-1,3,4-oxadiazol-2-yl)acetamide	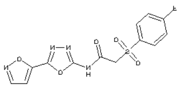	352,303	Amb1379066
**36**	(NZ)-N-[5-(methoxymethyl)-3H-1,3,4-thiadiazol-2-ylidene]-2-[(3R)-2-oxoindolin-3-yl]acetamide	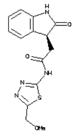	318,358	Amb22014468

Initially, 38 compounds were selected to dock to the ATP binding site of human PFK-P isoenzyme, of which 18 were commercially available and purchased from Enamine (Kyiv, Ukraine) (Inh, No, 1-8), ChemBridge (San Diego, USA) (Inh. No. 9-16) and ChemDiv (San Diego, CA) (Inh. No. 17-18). Because the human PFK-M (UniProt P08237-1) isoform has identical ATP-binding sites to the PFK-P isoform, the same compounds might equally inhibit both enzymes.

However, in one amino acid residue, human PFK-L's (UniProt P17858) ATP binding site differs from human PFK-P and human PFK-M isoenzymes. The model with a modified ATP binding site, where arginine at position 102 was substituted for threonine, was generated by using the most probable rotamer obtained from the Dunbrack database (http://dunbrack.fccc.edu/bbdep2010/) that exhibited no steric clashes. Distances among specific moieties on amino acid residues and ATP molecules at both ATP binding sites correspond to the interactions between the protein and ATP molecule (Figure 4a, b). Accordingly, an additional 15 potential PFK-L inhibitors were selected and purchased from Enamine (Kyiv, Ukraine) (Inh. No. 21-28) and ChemBridge (San Diego, CA) (Inh. No. 29-32, 34-36). The Inh. No. 33 was commercially unavailable.

All purchased compounds were supplied as 5mg lyophilized powder and dissolved in DMSO to the final concentration of 10 mM before use.
