# Supplementary material for: Small-molecule inhibitors of 6-phosphofructo-1-kinase simultaneously suppress lactate and superoxide generation in cancer cells
Source: PLoS One. 2025 May 21;20(5):e0321998. doi: 10.1371/journal.pone.0321998 (PMC12094722; doi:10.1371/journal.pone.0321998)
Supplement: S1 Fig — (PDF) [file pone.0321998.s004.pdf]

S1 Fig. Preliminary screening of selected compounds in colorectal adenocarcinoma Caco-2 cells.

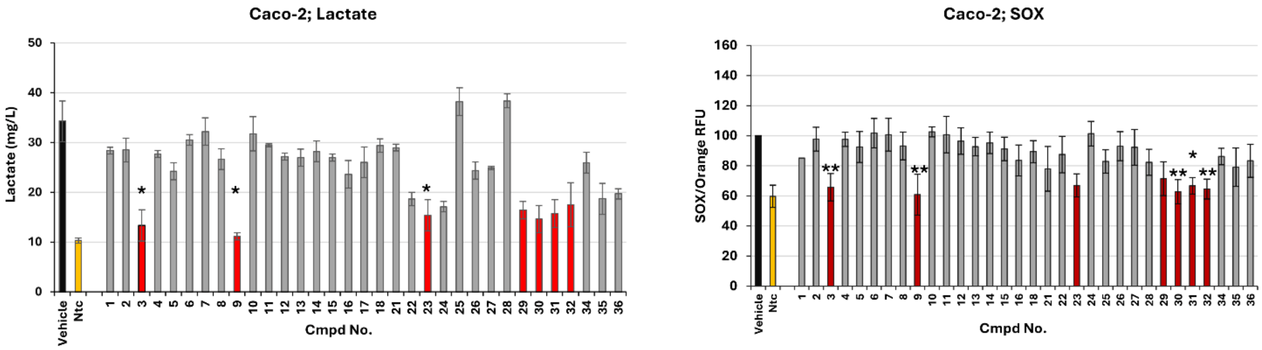

Preliminary screening for suppressing lactate formation in Caco-2 cells by testing selected compounds revealed four cmpds No. 3, 9, 23, and 30 to suppress lactate (P* <0,005) similarly, cmpds to the vehicle. Suppressing SOX formation and No. 3, 9, 30, and 32 proved more effective (P** <0.001) than the vehicle after 36 hours of incubation. Data represents three independent measurements as mean ±SD (n=3).
