# Supplementary material for: Small-molecule inhibitors of 6-phosphofructo-1-kinase simultaneously suppress lactate and superoxide generation in cancer cells
Source: PLoS One. 2025 May 21;20(5):e0321998. doi: 10.1371/journal.pone.0321998 (PMC12094722; doi:10.1371/journal.pone.0321998)
Supplement: S2 Fig — (PDF) [file pone.0321998.s005.pdf]

S2 Fig. Preliminary screening of selected compounds in melanoma COLO 829 cells.

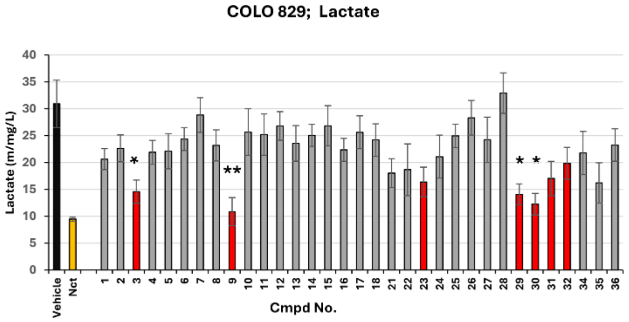

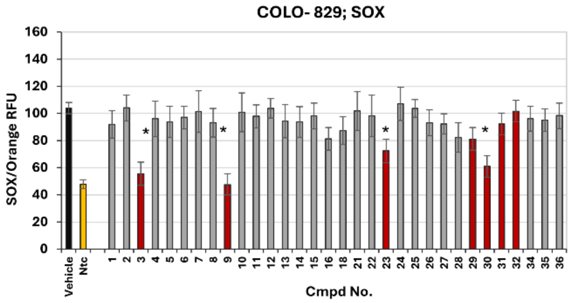

By screening 33 compound inhibitors for reducing lactate formation at COLO 829 cells, cmpds No. 3, 29, and 30 proved significantly efficient (P* <0.005) compared to the vehicle. In contrast, the efficiency of cmpds No. 9 was more substantial (P** <0,001), and SOX suppression was found at cmpds No. 3, 9, 23, and 30) (P* <0.005) compared to the vehicle after 36 hours of incubation. Data represents three independent measurements and are presented as mean SD (n=3).
