# Supplementary material for: Small-molecule inhibitors of 6-phosphofructo-1-kinase simultaneously suppress lactate and superoxide generation in cancer cells
Source: PLoS One. 2025 May 21;20(5):e0321998. doi: 10.1371/journal.pone.0321998 (PMC12094722; doi:10.1371/journal.pone.0321998)
Supplement: S3 Fig — (PDF) [file pone.0321998.s006.pdf]

S3 Fig. Preliminary screening of selected compounds in breast gland adenocarcinoma MDA-MD-231 cells.

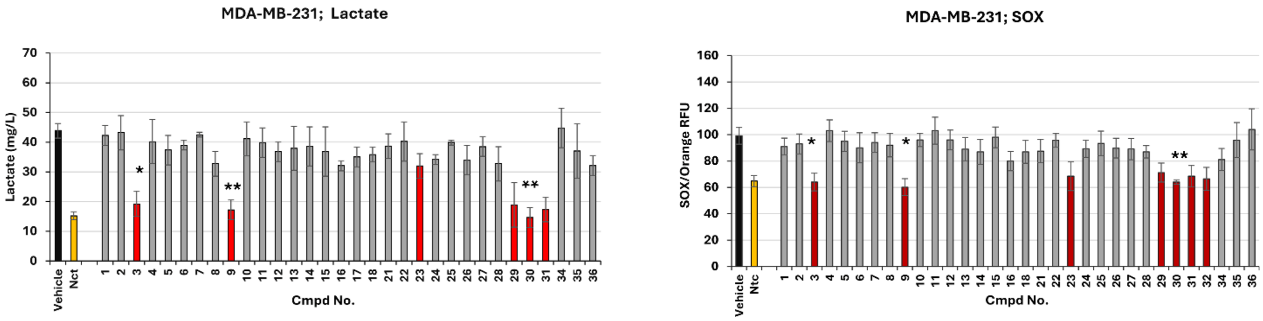

In the MDA-MB-231 cells, the lowest level of significant difference between lactate suppression between treated and untreated cells was observed by cmpds No. 9 and 30 (P** <0.001). In contrast, with cmpds No. 3, slightly higher levels were detected (P* <0.005). By measuring SOX suppression, cmpd No. 30 proved the most successful (P** value <0.001) compared to the vehicle after 36 hours of incubation. At the same time, cmpds No. 3 and 9 revealed slightly higher values (P* value < 0.005) by screening 33 compound inhibitors for reducing lactate and super-oxide formation Inh. No. 9 and 30 have shown the lowest significance values between vehicle and treated MDA-MB-231 cells of both tested metabolites; however, Inh. No. 3 successfully reduced lactate formation as well. Data represents three independent measurements and is presented as mean ±SD (n=3).
