# Supplementary figures and images for: Small-molecule inhibitors of 6-phosphofructo-1-kinase simultaneously suppress lactate and superoxide generation in cancer cells
Source: PLoS One. 2025 May 21;20(5):e0321998. doi: 10.1371/journal.pone.0321998 (PMC12094722; doi:10.1371/journal.pone.0321998)

**S4 Fig. Dose-dependent inhibition of lactate formation in Caco-2 cells.**

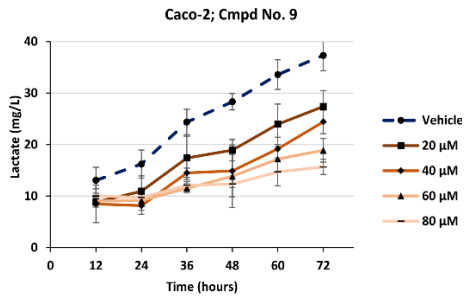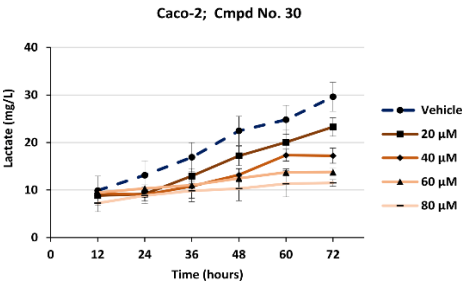

Supplement: S4 Fig — (PDF) [file pone.0321998.s007.pdf]
