# Supplementary material for: Small-molecule inhibitors of 6-phosphofructo-1-kinase simultaneously suppress lactate and superoxide generation in cancer cells
Source: PLoS One. 2025 May 21;20(5):e0321998. doi: 10.1371/journal.pone.0321998 (PMC12094722; doi:10.1371/journal.pone.0321998)
Supplement: S5 Fig — (PDF) [file pone.0321998.s008.pdf]

S5 Fig. Dose-dependent inhibition of lactate formation in Caco-2 cells – cytostatic effect.

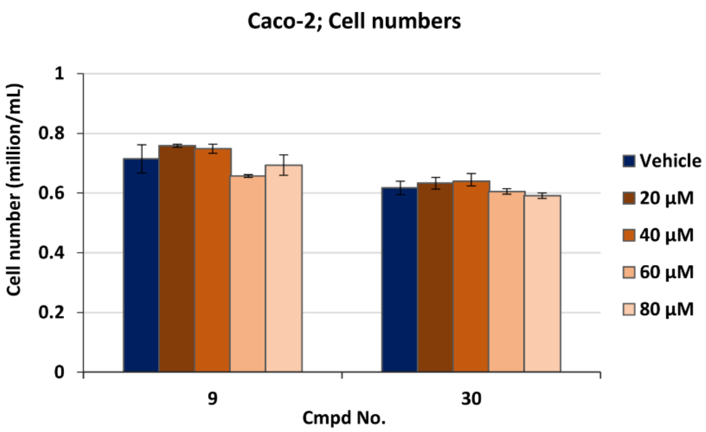

No cytostatic features could be observed by increasing the concentration of cmpds up to 80 µM after 72 hours of incubation.
