# Supplementary material for: Small-molecule inhibitors of 6-phosphofructo-1-kinase simultaneously suppress lactate and superoxide generation in cancer cells
Source: PLoS One. 2025 May 21;20(5):e0321998. doi: 10.1371/journal.pone.0321998 (PMC12094722; doi:10.1371/journal.pone.0321998)
Supplement: S6 Fig — (PDF) [file pone.0321998.s009.pdf]

S6 Fig. Dose-dependent inhibition of lactate formation in Caco-2 cells – cytotoxic effect.

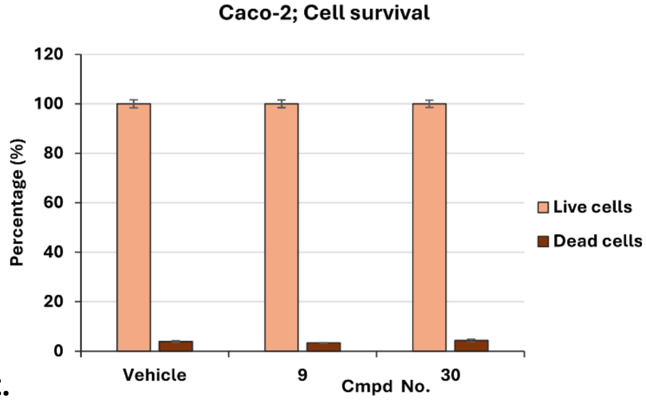

In Caco-2 cells no cytotoxic effects of the inhibitors were observed either. The average percentage of dead cells in control and the presence of the different concentrations of inhibitors was as follows: Vehicle (3.84±0.54%), and No, 9 (3.33±0.14%), and cmpd No. 30 (4.32±0.51%). Data are representative of independent measurement and are presented as mean ±SD (n-3).
