# Supplementary material for: Small-molecule inhibitors of 6-phosphofructo-1-kinase simultaneously suppress lactate and superoxide generation in cancer cells
Source: PLoS One. 2025 May 21;20(5):e0321998. doi: 10.1371/journal.pone.0321998 (PMC12094722; doi:10.1371/journal.pone.0321998)
Supplement: S9 Fig — (PDF) [file pone.0321998.s012.pdf]

S9 Fig. Dose-dependent inhibition of lactate formation in COLO cells – cytotoxic effect.

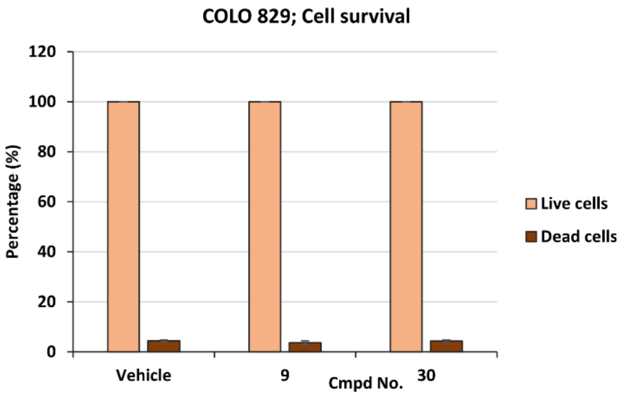

No significant cytotoxic effects of the inhibitors were observed. The average percentage of dead cells in control and the presence of the different concentrations of inhibitors was as follows: Vehicle (4.4±0.44%), cmpd No. 9 (3.65±0.78%), and cmpd No. 30 (4.27±0.49%). Data are presentative of independent measurement and presented as mean ±SD (n-3).
