# Supplementary material for: Small-molecule inhibitors of 6-phosphofructo-1-kinase simultaneously suppress lactate and superoxide generation in cancer cells
Source: PLoS One. 2025 May 21;20(5):e0321998. doi: 10.1371/journal.pone.0321998 (PMC12094722; doi:10.1371/journal.pone.0321998)
Supplement: S10 Fig — (PDF) [file pone.0321998.s013.pdf]

S10 Fig. Dose-dependent inhibition of lactate formation in MDA-MB-231.

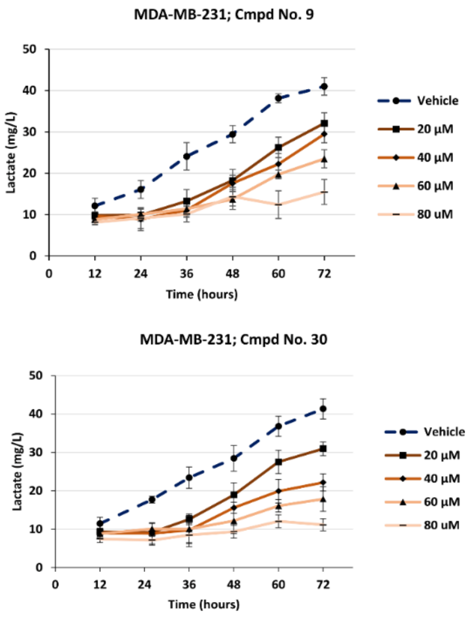

Dose-dependent effects of cmpds No. 9 and 30 on lactate suppression were observed, however, a solid, gradual decrease in the inhibition effect of both cmpds was observed after a certain period.
