# Supplementary material for: Small-molecule inhibitors of 6-phosphofructo-1-kinase simultaneously suppress lactate and superoxide generation in cancer cells
Source: PLoS One. 2025 May 21;20(5):e0321998. doi: 10.1371/journal.pone.0321998 (PMC12094722; doi:10.1371/journal.pone.0321998)
Supplement: S12 Fig — (PDF) [file pone.0321998.s015.pdf]

**S12 Fig. Dose-dependent inhibition of lactate formation in MDA-MB-231 cells – cytotoxic effect.**

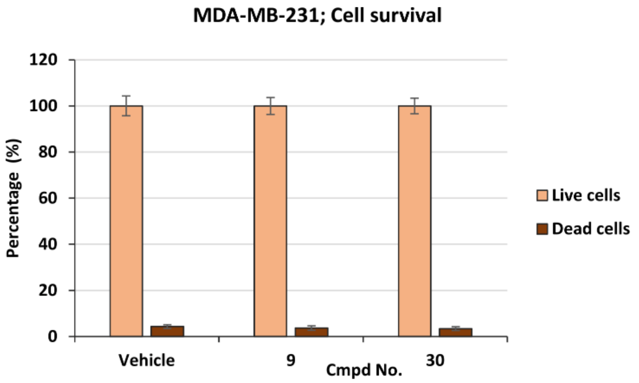


No significant differences in total cell numbers were observed due to the different concentrations of inhibitors in the medium. The average percentage of dead MDA-MB-231 cells in the control and the presence of the inhibitors was as follows: Vehicle (4.28±0.78%), cmpd No. 9 (6.59±0.29%), and cmpd No. 30 (3.4±0.21%). Data are presentative of independent measurement and presented as mean ±SD (n-3).
