# Supplementary material for: Small-molecule inhibitors of 6-phosphofructo-1-kinase simultaneously suppress lactate and superoxide generation in cancer cells
Source: PLoS One. 2025 May 21;20(5):e0321998. doi: 10.1371/journal.pone.0321998 (PMC12094722; doi:10.1371/journal.pone.0321998)
Supplement: S13 Fig — (PDF) [file pone.0321998.s016.pdf]

S13 Fig. Lactate suppression by sequential re-insertion of inhibitors at low concentration in Caco-2 cells.

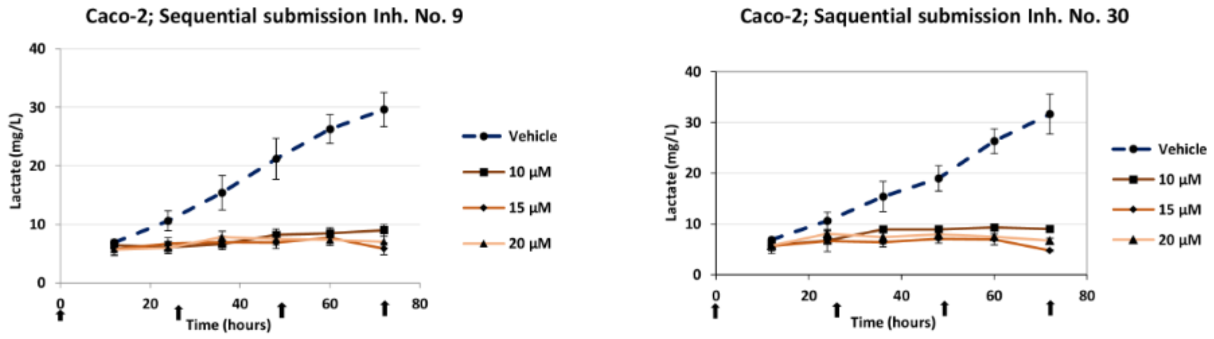

Significantly reduced lactate generation was detected in Caco-2 cells if cmpds No. 9 and 30) were periodically added to the medium at low concentrations (10, 15, and 20 μM) every 24 hours. Statistically significant differences measured at the end of incubation (72 hours) between the vehicle and cells treated with 10 μM of camps No. 9 and No. 30 have P values <0.0005 and P<0.0001, respectively. No significant cytotoxic effects of the inhibitors were observed after 72 hours of incubation, although different concentrations of inhibitors were added sequentially. The following average percentages of dead cells were observed in the medium without added inhibitors (4.45±0,41%) and with the cells sequentially treated with 10 μM cmpd No. 9 (4.03±0.32%), and cmpd No. 30 (4.24±0.38%). The data represent three independent measurements and are presented as mean ±SD (n=3).
