# Supplementary material for: Small-molecule inhibitors of 6-phosphofructo-1-kinase simultaneously suppress lactate and superoxide generation in cancer cells
Source: PLoS One. 2025 May 21;20(5):e0321998. doi: 10.1371/journal.pone.0321998 (PMC12094722; doi:10.1371/journal.pone.0321998)
Supplement: S14 Fig — (PDF) [file pone.0321998.s017.pdf]

S14 Fig. Lactate suppression by sequential re-insertion of inhibitors at low concentration in COLO 829 cells.

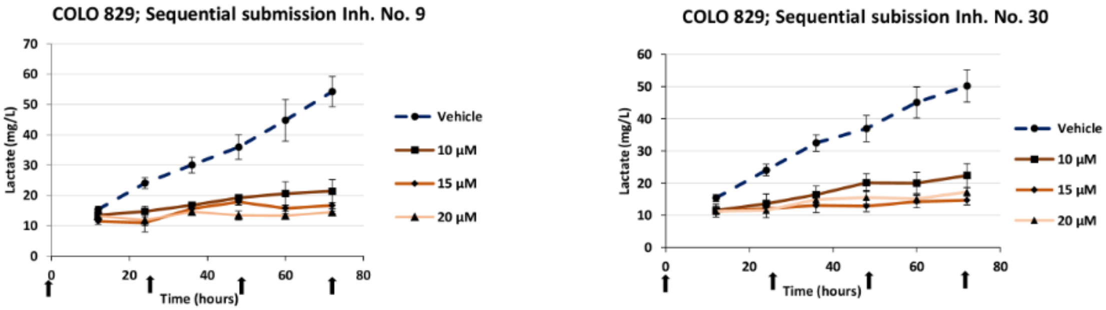

Reduced lactate generation was also detected in COLO-829 cells if inhibitors (cmpd No. 9 and 39) were periodically added to the medium at low concentrations (10, 15, and 20 μM) every 24 hours. However, a minor lactate generation was observed by 10 μM concentration of both tested inhibitors. Statistically significant differences measured at the end of incubation (72 hours) between the vehicle and cells treated with 10 μM of cmpds No. 9 and No. 30 have P values <0.001 and P <0.005, respectively. After 72 hours of incubation, no significant cytotoxic effect of the inhibitors could be detected, although different concentrations of inhibitors were added sequentially. The following average percentages of dead cells were observed in the medium without added inhibitors (2.44±0.25%) and with the cells sequentially treated with 10 μM cmpd No. 9 (2.84±0.19%) and cmpd No. 30 (2.42±0.21%). The data represent three independent measurements and are presented as mean ±SD (n=3).
