# Supplementary material for: Small-molecule inhibitors of 6-phosphofructo-1-kinase simultaneously suppress lactate and superoxide generation in cancer cells
Source: PLoS One. 2025 May 21;20(5):e0321998. doi: 10.1371/journal.pone.0321998 (PMC12094722; doi:10.1371/journal.pone.0321998)
Supplement: S15 Fig — (PDF) [file pone.0321998.s018.pdf]

S15 Fig. Lactate suppression by sequential re-insertion of inhibitors at low concentration in MDA-MB-231 cells.

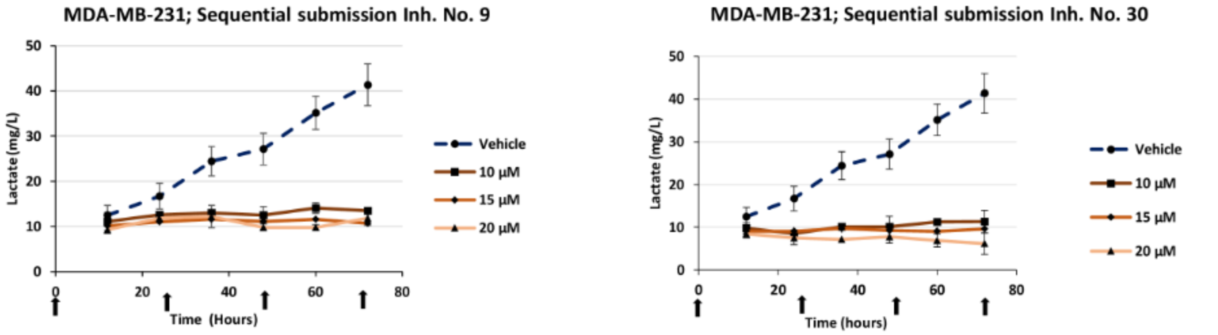

Significantly reduced lactate generation was detected in MDA-MB-231 if inhibitors (cmpd No. 9 and 39) were periodically (24 hours) added to the medium at low concentrations (10, 15, and 20 µM) every 24 hours. Statistically significant differences measured at the end of incubation (72 hours) between the vehicle and cells treated with 10 µM of cmpds No. 9 and No. 30 have P values of <0.001 and <0.0005, respectively. Similar results of suppressed lactate formation with inhibitors were obtained in **MDA-MB-231** cells. No significant cytotoxic effect of the inhibitors could be detected after 72 hours. The following average percentages of dead cells were observed in the medium without added inhibitors (2.52±0.356%) and with the cells sequentially treated with 10 µM inhibitor No. 9 (2.35±0.09%) and inhibitor No. 30 (2.32±0.21%). The data represent three independent measurements and are presented as mean ±SD (n=3).
