# Supplementary material for: Small-molecule inhibitors of 6-phosphofructo-1-kinase simultaneously suppress lactate and superoxide generation in cancer cells
Source: PLoS One. 2025 May 21;20(5):e0321998. doi: 10.1371/journal.pone.0321998 (PMC12094722; doi:10.1371/journal.pone.0321998)
Supplement: S16 Fig — (PDF) [file pone.0321998.s019.pdf]

S16 Fig. Superoxide (SOX) and reactive oxygen species (ROS) suppression by sequential re-insertion of inhibitors at low concentrations in Caco-2 cells.

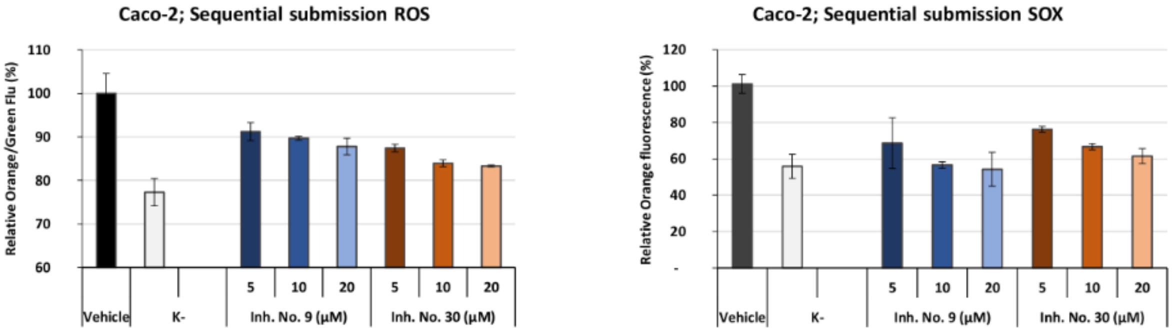

Suppressed ROS and SOX generations were detected in Caco-2 cells when inhibitor No. 9 or 30 was sequentially re-inserted into the medium at low concentrations (5, 10, and 15 µM) every 24 hours. Strong preventions of SOX formation were detected, while less significant differences between the treated and untreated cells (vehicle) were observed in ROS measurements. The values of statistically significant differences conducted as described before were as follows: ROS cmpds No. 9 ($P < 0.5$), and No 30 ($P < 0.1$); SOX cmpd No. 9 ($P < 0.005$) and No. 30 ($P < 0.005$).
