# Supplementary material for: Small-molecule inhibitors of 6-phosphofructo-1-kinase simultaneously suppress lactate and superoxide generation in cancer cells
Source: PLoS One. 2025 May 21;20(5):e0321998. doi: 10.1371/journal.pone.0321998 (PMC12094722; doi:10.1371/journal.pone.0321998)
Supplement: S17 Fig — (PDF) [file pone.0321998.s020.pdf]

S17 Fig. Superoxide (SOX) and reactive oxygen species (ROS) suppression by sequential re-insertion of inhibitors at low concentrations in COLO 829 cells.

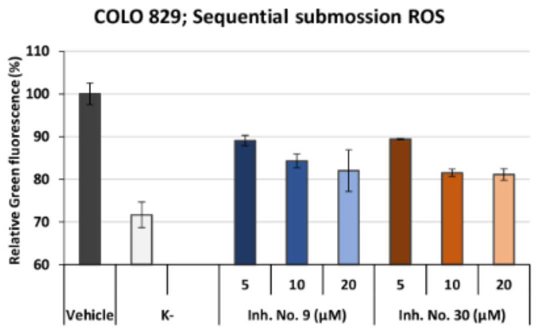

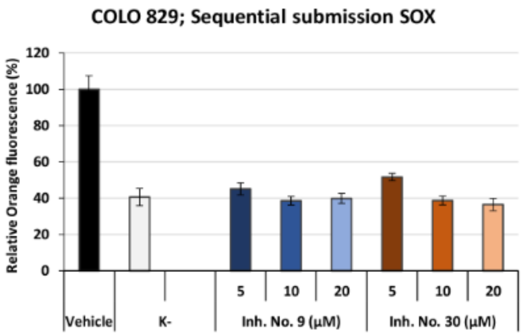

Suppressed ROS and SOX generations were detected in COLO 829 cells when inhibitor No. 9 or 30 was sequentially re-inserted into the medium at low concentrations (5, 10, and 15 µM) every 24 hours. Somehow, stronger preventions of ROS and SOX formation were detected by COLO cells compared to other tested tumorigenic cells. The values of statistically significant differences between treated and untreated cells conducted as described before, were as follows: ROS cmpds No. 9 ($P < 0.01$), and No. 30 ($P < 0.005$); SOX cmpds No. 9 ($P < 0.001$) and No. 30 ($P < 0.001$).
