# Supplementary material for: Small-molecule inhibitors of 6-phosphofructo-1-kinase simultaneously suppress lactate and superoxide generation in cancer cells
Source: PLoS One. 2025 May 21;20(5):e0321998. doi: 10.1371/journal.pone.0321998 (PMC12094722; doi:10.1371/journal.pone.0321998)
Supplement: S18 Fig — (PDF) [file pone.0321998.s021.pdf]

S18 Fig. Superoxide (SOX) and reactive oxygen species (ROS) suppression by sequential re-insertion of inhibitors at low concentrations in MDA-MB-231 cells.

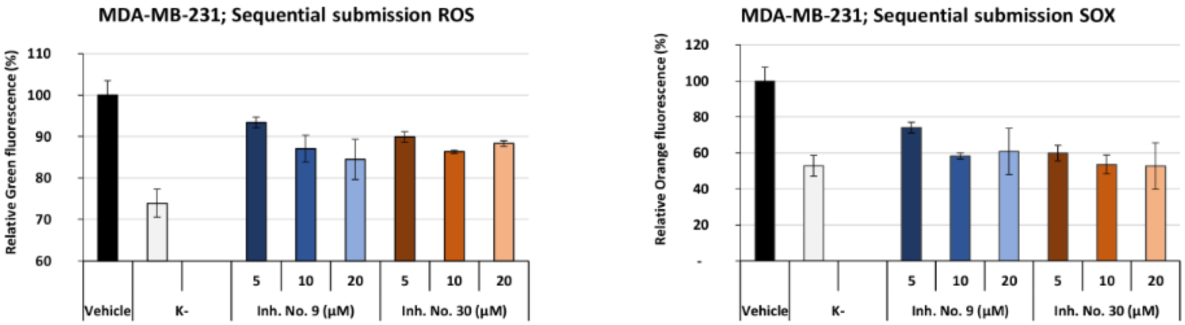

Suppressed ROS and SOX generations were detected in MDA-MB-231 cells when inhibitor No. 9 or 30 was sequentially re-inserted into the medium at low concentrations (5, 10, and 15 µM) every 24 hours. Again, strong preventions of SOX formation were detected, while less significant differences between the treated and untreated cells (vehicle) were observed in ROS measurements. The values of statistically significant differences conducted as described before were as follows: ROS cmpdc No. 9 ($P < 0.5$, and No. 30 ($P < 0.05$); SOX cmpdc No. 9 ($P < 0.01$) and No. 30 ($P < 0.005$).
