# Supplementary material for: Small-molecule inhibitors of 6-phosphofructo-1-kinase simultaneously suppress lactate and superoxide generation in cancer cells
Source: PLoS One. 2025 May 21;20(5):e0321998. doi: 10.1371/journal.pone.0321998 (PMC12094722; doi:10.1371/journal.pone.0321998)
Supplement: S19 Fig — (PDF) [file pone.0321998.s022.pdf]

S19 Fig. The graphs for OCR and ECAR calculations on Jutkat cells were analyzed in time.

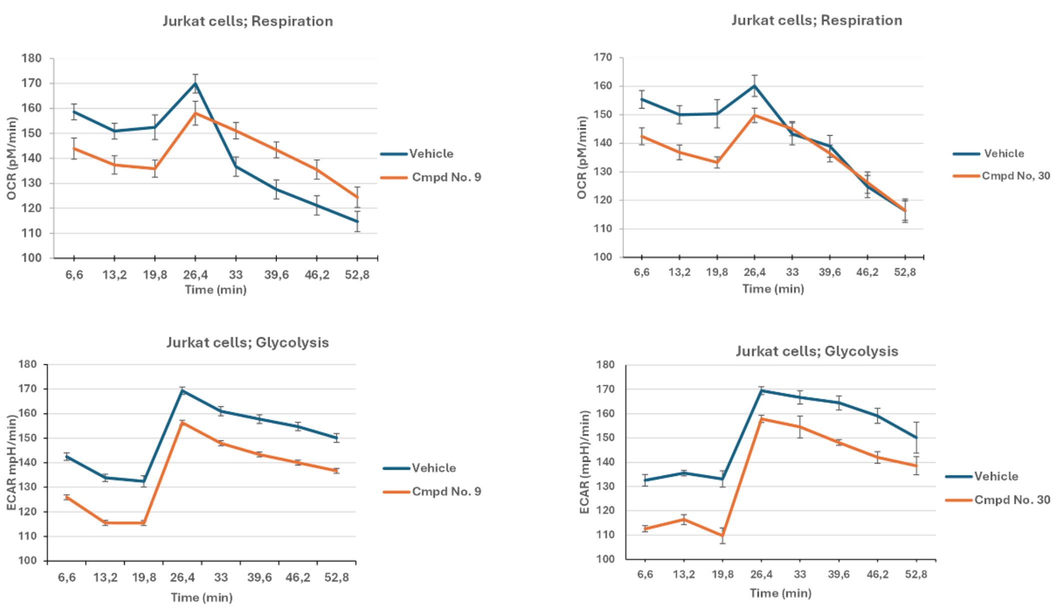

Respiratory fluxes, maximal respiration rates, glycolytic rates, and glycolytic capacities were measured in Jurkat cells in the presence and absence of selected cmpds No. 9 and No. 30. To get more information about the measurement, the graphs of in-time analysis are shown below. Twenty minutes after the start of the experiment, 1μM of Oligomycin A and 0.25 μM of FCCP were added to the medium. The measurements of OCR and ECAR in the medium were conducted just before the addition of Oligomycin A and FCCP to measure essential respiration and glycolysis. Next, measurements were performed for about 7 minutes after adding added reagents to calculate maximal respiration and glycolytic capacity. Data represents three independent measurements and is presented as mean ±SD (n=3).
